# Key defatting tissue pretreatment protocol for enhanced MALDI MS Imaging of peptide biomarkers visualization in the castor beans and their attribution applications

**DOI:** 10.3389/fpls.2022.1083901

**Published:** 2022-12-16

**Authors:** Luyuan Qin, Junshan Han, Chuang Wang, Bin Xu, Deyun Tan, Song He, Lei Guo, Xiaochen Bo, Jianwei Xie

**Affiliations:** ^1^ State Key Laboratory of Toxicology and Medical Countermeasures, Institute of Pharmacology and Toxicology, Academy of Military Medical Sciences, Beijing, China; ^2^ Department of Bioinformatics, Institute of Health Service and Transfusion Medicine, Beijing, China; ^3^ Ministry of Education Key Laboratory of Ethnic Medicine, College of Pharmacy, Minzu University of China, Beijing, China; ^4^ Institute of Cash Crop Research, Zibo Academy of Agricultural Sciences, Zibo, China

**Keywords:** castor beans, MALDI-MSI, tissue washing, peptide attribution markers, geographical origin

## Abstract

**Introduction:**

Castor bean or ricin-induced intoxication or terror events have threatened public security and social safety. Potential resources or materials include beans, raw extraction products, crude toxins, and purified ricin. The traceability of the origins of castor beans is thus essential for forensic and anti-terror investigations. As a new imaging technique with label-free, rapid, and high throughput features, matrix-assisted laser desorption ionization mass spectrometry imaging (MALDI-MSI) has been gradually stressed in plant research. However, sample preparation approaches for plant tissues still face severe challenges, especially for some lipid-rich, water-rich, or fragile tissues. Proper tissue washing procedures would be pivotal, but little information is known until now.

**Methods:**

For castor beans containing plenty of lipids that were fragile when handled, we developed a comprehensive tissue pretreatment protocol. Eight washing procedures aimed at removing lipids were discussed in detail. We then constructed a robust MALDI-MSI method to enhance the detection sensitivity of RCBs in castor beans.

**Results and Discussion:**

A modified six-step washing procedure was chosen as the most critical parameter regarding the MSI visualization of peptides. The method was further applied to visualize and quantify the defense peptides, Ricinus communis biomarkers (RCBs) in castor bean tissue sections from nine different geographic sources from China, Pakistan, and Ethiopia. Multivariate statistical models, including deep learning network, revealed a valuable classification clue concerning nationality and altitude.

## 1 Introduction

As one of the top ten oil crops in the world, the castor oil plant, *Ricinus communis*, is originated in Africa and currently widely distributed in tropical, subtropical, and temperate regions worldwide (Polito et al., 2019). It has reached an industrial scale in more than 30 countries because of its high economic value. At least one million tons of castor beans are harvested and processed annually to prepare castor oil, a widely used raw material in mechanical and chemical industries, cosmetics, and pharmaceutical industries. However, castor bean contains a relatively high abundance of water-soluble toxic protein, ricin, which is easily extracted into aqueous solutions during castor oil production.

Due to easy preparation, strong concealment, vast source, and no specific antidote, ricin has had military, criminal and terrorist uses in history. It is one of the typical “culprits” in terror events or toxication incidents. A notorious case of intoxication was the death of Georgi Markov in 1978, and ricin was identified as the agent used for his assassination (Darby et al., 2001). In 2002, castor beans and the method for preparing ricin were discovered during a raid on terrorists in London. Since then, a series of ricin terror incidents have occurred. From 2012 to 2018, ricin letters were intermittently sent to the White House, causing great panic in the international community. Accidental oral ingestion or intentional poisoning *via* castor beans or defatted press cake fertilizer has occurred from time to time, either for human beings or livestock (Worbs et al., 2011; Aggarwal et al., 2017).

Castor beans, raw extraction products, crude toxins and purified ricin are all potential resources or materials in the intoxication or bioterror events left for forensic and anti-terrorism investigation. In response to the national security, public security, and clinical toxication caused by castor beans originating from varied resources and locations worldwide, the traceability of castor beans from different cultivars and geographic locations is a critical issue addressed.

How could we quickly obtain the proof of origin or accomplished cultivar identification when we deal with castor beans as one of the main pieces of evidence in forensic science? Peptide mass fingerprinting information of digested *Ricinus communis* extracts, or ricin, ricin agglutinin provided by MALDI-MS (Duriez et al., 2008) was a helpful approach. In addition to ricin itself, other characteristic components in castor beans are expected to provide an alternative way. For example, Darby et al. identified the alkaloid ricinine in castor beans as a co-existed substance with ricin based on liquid chromatography-mass spectrometry (LC-MS) and matrix-assisted laser desorption/ionization-time of flight mass spectrometry (MALDI-TOF MS) (Darby et al., 2001). Pigott et al. focused on analyzing the metabolome of castor beans for cultivar and provenance determination by ^1^H-Nuclear magnetic resonance spectroscopy (Pigott et al., 2011). Therefore, the direct detection of low molecular weight biomarkers in castor beans may be a faster, more effective, and more reliable alternative method, which can aid in the cultivar and geographic source determination of castor beans (Vuckovic et al., 2016).

Besides ricin, ricinine, and the metabolomes, Ovenden (Ovenden et al., 2009) et al. identified three low molecular weight peptides in six different cultivars of castor beans by LC-MS and MALDI-MS, known as *Ricinus communis* biomarkers or RCBs, designated as RCB-1, -2, and -3, respectively. RCB-1 and RCB-2 were commonly found in varying amounts in six different cultivars, while RCB-3 was found only in the “*Carmencita*” cultivar. After that, Ovenden et al. identified RCB-4 and RCB-5 in the castor beans of the “*impala*” cultivar (Ovenden et al., 2014). Moreover, Fredriksson et al. (Fredriksson et al., 2018) developed a method for chemical analysis of forensic attribution markers related to the purification of ricin based on a complex set of biomarkers including carbohydrates, fatty acids, RCBs, ricin, etc. (Fredriksson et al., 2018). Compared with the toxic protein, peptides have better sensitivity and easier pretreatment due to their more straightforward structure. This previous research revealed the potential of RCBs as attribution biomarkers compounds in distinguishing the cultivars of castor beans. It inspires our interest in unveiling the inherent information of this series of peptides with more advanced and fully informative techniques.

Mass spectrometry imaging (MSI) is a label-free molecular imaging technique with simple sample preparation, which can identify molecular compositions in biological samples and visualize the spatial distribution of compounds *in situ* in tissue section and is a powerful non-targeted analysis tool. The most widely used ionization method in MSI is MALDI, a soft ionization mass spectrometry technique with less fragmentation, high spatial resolution, sensitivity, and imaging speed (Greco et al., 2018; Neagu et al., 2019). With the prosperous and continuous development, MALDI-MSI has been gradually applied in the field of plant research in recent years, such as revealing the spatial distribution of metabolites in *Clausena lansium* tissue sections (Tang et al., 2021) and *in situ* characterizing peptides in *Medicago truncatula* root nodules (Keller et al., 2020) as well as proteins in tomato (Bencivenni et al., 2014), etc. Recently, Sturtevant et al. revealed heterogeneous tissue distribution of mono-, di- and tri-hydroxy-triacylglycerols in the embryo and endosperm tissues of castor beans to understand better the regulation of triacylglycerol accumulation in oilseeds by MALDI-MSI (Sturtevant et al., 2019).

Many endogenous plant peptides can be served as significant markers of plant growth and development, defense responses, and symbiotic relationships with other species. It is hence increasingly necessary to reveal the role of endogenous peptides at the molecular level. Gemperline (Gemperline et al., 2016) et al. uncovered the spatial distribution of hundreds of endogenous peptides in different growth stages of legume *Medicago truncatula* by MALDI-MSI. They speculated the transfer of endogenous peptides through the distribution differences in seedlings, mature roots, and nodules. It thus revealed the potential of MALDI-MSI towards endogenous plant peptide markers.

In MALDI-MSI, the optimization of sample preparation is the most crucial step to ensure high sensitivity, high spatial resolution, high-quality signal, and intensity. However, compared to animal tissues, sample preparation approaches are more challenging for plant tissues due to the inherent features of plants, especially for some lipid-rich, water-rich, or fragile plant tissues. Therefore, developing a specific optimized sample preparation protocol is essential for MALDI-MSI detection of peptides in plants, such as obtaining a uniform matrix spray effect and optimizing washing steps. Several works have confirmed that the action of washing sections with organic solvents, such as ethanol, isopropanol, toluene, xylene, and chloroform, etc., prior to matrix application and MALDI-MSI is effective to remove plenty of the lipids and salts, thereby increasing the intensity of peptides and proteins (Goodwin et al., 2008; Thomas et al., 2013; Buchberger et al., 2020; Vu et al., 2021). But most of them focused on animal tissue sections, and no reports on lipid removal from lipid-rich plant tissue sections can be found. Therefore, the washing protocol for lipid removal should be thoroughly investigated for this type of sample.

In this paper, we proposed a systematic sample preparation protocol for castor beans containing plenty of lipids, and their tissue sections were fragile when handled. The washing protocols of lipid removal were focused on, with the aid of staining and MSI visualization of peptides. A modified six-step washing procedure was finally chosen among all eight measures to remove lipids as much as possible. We then constructed a MALDI-MSI method to enhance the detection sensitivity of defense peptides, RCB-1, RCB-2, and RCB-3, in castor beans. We further established a stable isotope internal standard (IS) quantitative method of MALDI-MSI to determine the content of RCBs in castor beans. Moreover, regarding the distribution of different geographic sources revealed by the defatted MSI signals in castor beans, we employed several statistical and classification tools to unravel their characteristics. We hope this work will provide a new research perspective for the traceability of castor bean relevant events of castor bean intoxication.

## 2 Materials and methods

### 2.1 Reagents and materials

2,5-dihydroxybenzoic acid (DHB) and α-Cyano-4-hydroxycinnamic Acid (CHCA) were purchased from Brucker Daltonik GmbH (Bremen, Germany). Sodium salt of carboxy methyl cellulose (CMC-Na) was purchased from Sigma-Aldrich Co. (USA). Acetonitrile (ACN), methanol, ethanol, isopropanol, methylbenzene, dimethylbenzene, and trifluoroacetic acid (TFA) were obtained from Merck (Melbourne, Australia). Peptide calibration standard II (pep II), designed for calibration in a low mass range between 700 and 3200 Da, was purchased from Brucker Daltonik GmbH (Bremen, Germany). Standard peptides of RCB-1, RCB-2, and RCB-3 were synthesized by Sangon Biotech with a HPLC purity of 98.9% (Shanghai, China). Stable-isotopically labeled internal standard (SILIS) of RCB-2 was synthesized by Synpeptide Co. (Nanjing, China), the carbon and nitrogen atoms in the twelfth phenylalanine in the amino acid sequence were labeled (^13^C_9_^15^N_1_-RCB-2). All reagents and solvents used in this work were of analytical grade or higher. Ultrapure water was generated by a Milli-Q A10 water purification system (Millipore, MA, USA).

Resistance conductive indium tin oxide (ITO)-coated microscope glass was purchased from Brucker Daltonik GmbH (Bremen, Germany). Oil Red O Staining Kit was purchased from Beyotime (Shanghai, China). The castor beans (*Ricinus communis*) originated from Beijing, Xinjiang Uygur Autonomous Region, Hebei, Shanxi, Inner Mongolia Autonomous Region, and Sichuan provinces in China were purchased from the local medicine markets. The castor beans originated from Shandong (commercial brand Zibi No. 9), Ethiopia in Africa, and Pakistan in Asia were kindly provided by Zibo Academy of Agricultural Sciences. The species of castor beans were identified by Prof. Deyun Tan.

### 2.2 Instruments

Autoflex III MALDI TOF/TOF MS instrument (Bruker Daltonik, Germany), in which a Smartbeam 3D Nd: YAG (355 nm) at a frequency of 200 Hz was mounted, FlexControl 3.4 software for acquisition, and FlexImaging 4.1 for MSI data collection (Bruker Daltonik, Germany). Cryostat sectioning was performed in a CM 1950 cryostat (Leica, Germany), and optical scanning was finished with a Perfection V370 scanner (EPSON, Japan). The matrix spraying or coating was performed in a GET-Sprayer (Beijing Huayi Innovation &Biotechnology Co., Beijing, China).

### 2.3 Sample preparation

A sample preparation optimization procedure was carried out, including matrix solution composition, tissue washing and matrix coating. The sample was visually evaluated after each step of sample preparation with the CCD camera inside the Autoflex III MALDI-MS during MSI measurements.

#### 2.3.1 Tissue sectioning

The castor beans were peeled to remove the outer seed coat and then embedded in the prepared 4% CMC-Na solution. The CMC-Na-embedded seeds were frozen at -20°C for five days before sectioning. Seeds were cryo-sectioned at -30°C into 20 μm thickness and immediately thaw-mounted on the conductive sides of ITO-coated microscope glass slides. The slides were dried under a vacuum for 20 minutes and stored at -80°C.

#### 2.3.2 Washing protocol

Before matrix coating, tissue sections were submerged into 20 mL of freshly prepared washing solvents for the time indicated in the specific protocol to remove lipids. Altogether, eight washing protocols were evaluated, and a “no wash” procedure was used as a negative control.

#### 2.3.3 Optimization of matrix solution composition

The concentration of CHCA, ACN, and TFA were the variables selected to be optimized for the matrix solution composition. The appropriate range of the three variables was determined based on the results of the single factor experiment. The box-Behnken design combined with response surface methodology (RSM) was performed to optimize the optimal CHCA matrix solutions for RCBs. Experimental design and result analysis were finished using Design-Expert 13.0.1.0 (Stat-Ease, Inc., Minneapolis, MN, USA).

#### 2.3.4 Matrix coating and optimization

Tissue section slices were evenly sprayed with CHCA matrix solutions using a GET-Sprayer. In the GET-Sprayer device, a high-voltage power source was added between the matrix solution sprayer system and the tissue section to form a strong formation between the spray needle and the conductive glass slide to obtain a uniformly distributed matrix coating effect. An orthogonal experimental design method was performed to establish the optimized matrix spraying conditions, in which the factors included air pressure, spraying flow rate, and spraying time.

### 2.4 MALDI-MSI analysis

Before MALDI-MSI, the instrument was calibrated by spotting the pep II calibration standard onto the matrix-coated ITO glass slide. All the mass spectra were automatically acquired over a mass range of m/z 1000 to 4000 in the positive reflection mode. Laser shots of 400 times were accumulated at one raster position and a raster step size of 100 µm.

### 2.5 Quantification of RCBs

For quantitative determination, a series of concentrations of RCB-1 to RCB-3 peptide standard mixture, including 100, 200, 500, 1000, and 5000 μg/L, were prepared in 50% ACN. Before matrix application, 1 µL of each of these solutions were deposited onto the blank area of the ITO slide around the tissue and air-dried. Aliquots of 500 μg/L SILIS of RCB-2 were uniformly mixed with CHCA matrix and then sprayed on the tissue sections and ITO slide. Calibration curves were generated by plotting the average intensity ratio of the major [M+H]^+^ ion of RCB-1 (m/z 2066.95), RCB-2 (m/z 1979.92), RCB-3 (m/z 1961.96) to the intensity of major [M+H]^+^ ion of ^13^C_9_^15^N_1_-RCB-2 (m/z 1989.92) versus the concentration of RCB-1 to 3 applied to the ITO slide (3 microspots for each concentration). Finally, the concentrations of RCBs in tissue sections from different origins were calculated by calibration curves.

### 2.6 Data reprocessing and multivariate statistical analysis

Phenotypes of five important agronomic traits of castor beans of nine geographical origins, including hundred-grain weight (one hundred random seeds for weighing were randomly selected), seed length (five seeds with the same size were randomly selected, the total length of five seeds head to tail were measured and the average value was taken), seed width (five seeds with the same size were randomly selected, the total length of five seeds from left to right were measured and the average value was taken), seed size (the seed length times the seed width was calculated as an index represented the overall size of the seed), and seed plumpness (the grain weight divided by the size, an index represented the character of the heaviness of seed endosperm) were statistically analyzed by GraphPad Prism 9.0.0. The violin plot was drawn to visually compare the phenotype and regional features of castor beans from different geographical sources. The dimension reduction of five agronomic traits data of castor bean samples from nine geographical origins was analyzed using the principal component analysis (PCA) model in OriginPro 2021 (OriginPro Co., USA).

The MSI data was visualized using FlexImaging v.4.1. Afterwards, the data were reprocessed and analyzed by SCiLS Lab version 2016b (Bruker, Germany), in which the normalization was performed based on the total ion count, and the PCA was applied for unsupervised analysis. The number of principal components was the minimum, with a cumulative contribution rate greater than 90%.

All MSI data were further analyzed by deep neural network (DNN, Spyder-python 3.9), in which the dual-channel DNN model was used to train the MSI data from nine different geographical sources for supervised analyses, including two types of characteristics, peak intensity and pixel position on tissue sections. The total sample data were obtained by summarizing the MSI data from nine different geographical sources and aligning the mass spectra peaks. All data were split into training, validation, and test sets at the ratio of 8:1:1, and the accuracy rate was used as the performance evaluation index. In the dual-channel DNN model, the total dimension of all features was 2065, the number of neurons in hidden layers of the sub-network where the peak intensity features were located was 512 and 256, and the number of neurons in output layer was 128. The number of neurons in hidden layers of the sub-network where the pixel features were located was 256 and 64, and the number of neurons in output layer was 1. After concatenating the outputs of the two sub-networks a 129-dimensional vector, which was then fed into a two-layer fully connected network (the third sub-networks) was obtained, and the number of neurons in the output layer was 9. The activation function was the ReLU function. Dropout and BatchNorm2d regularization were used to help prevent overfitting of the training. The loss function was the cross-entropy loss function, and the batch size was 10. Finally, the Shapley Additive explanations (SHAP) were used to interpret the classification results of the dual-channel DNN model. The core idea of SHAP was to calculate the marginal contribution of features to the model output. Distributed stochastic neighbor embedding (t-SNE) model was used to reduce the dimension of the output of the first layer of the fully connected network (the third sub-networks).

## 3 Results and discussion

### 3.1 Sample preparation

#### 3.1.1 Tissue sectioning

Note that the successful preparation of tissue sections is the first step but the most key parameter in imaging analysis. However, cryosection of fragile and high lipid plant tissues is quite challenging, and we should take care to avoid a critical loss of morphological features and delocalization (Li et al., 2018). The investigated objects in this work are the castor beans, which are presented as plant seeds with high lipid content, it is prone to curling and adhesion during the preparation of frozen tissue sections. Therefore, according to experience, 4% CMC-Na was used as embedding media to give adequate sample support and maintain tissue section morphology during cryo-sectioning. In addition, we found that for these lipid-rich plant seeds, entirely freezing the sample before slicing facilitated the preparation of topographically intact slices while keeping the blade temperature at -30 °C or lower. Finally, we prepared the castor bean sections with a thickness of 20 μm and intact tissue morphology.

#### 3.1.2 Effect of matrix solution composition on MSI

We firstly compared two most common matrices, DHB and CHCA for peptide measurement in MALDI and MALDI-MSI (Blutke et al., 2020) in the preliminary experiments. We chose CHCA as the matrix instead of DHB because had shown that CHCA was better than DHB in the detection of RCBs for providing stronger intensity and uniform crystallization (data not shown). Considering that the optimization of CHCA matrix solution composition for RCBs is necessary for good profiling and imaging data acquisition, we adopted RSM, the most relevant multivariate technique, for this analytical optimization. It describes the behavior of data sets to make accurate statistical previsions (Bezerra et al., 2008). Hereby we designed three experimental variables, including the concentration of CHCA, ACN, and TFA, respectively, to determine the optimized condition *via* RSM. According to the appropriate range of CHCA, ACN, and TFA concentration determined as 3~10 mg/mL, 50%~90%, and 0.1%~0.5%, respectively, by single factor experiment (**Supplemental Figure S1A**), the levels and actual values of parameters were settled (**Table S1**).

Experimental and predicted results of 17 randomized runs for selected responses obtained under different experimental conditions were presented in **Table S2**. It is shown that the predicted intensity values of RCB-1 to RCB-3 well matched the experimental results. Analysis of variance (ANOVA) for the response surface quadratic model of RCB-2 was adopted to assess the statistical significance of model coefficients. The significant model (p<0.05) and the non-significant lack of fit (p>0.05) validated the adequacy of the model that can be used for the optimization of the matrix solution composition (**Table S3**). ANOVA for the response surface quadratic model of RCB-1 and RCB-3 was also performed, and the influence of parameters was almost similar. The three-dimensional response plots and contour plots of RCB-1 to RCB-3 showed the interactive effect of investigated three variables (**Supplemental Figures S1B–D**). The optimized combination was finally set as 9 mg/mL CHCA dissolved in 90% aqueous ACN containing 0.1% TFA.

#### 3.1.3 Effect of matrix coating on MSI

In addition to matrix solution optimization, matrix coating for homogeneous matrix crystals is critical to obtain high-quality MALDI-MSI results. Among all matrix coating methods such as spray gun, sublimation, vibration spray, and electrospray ways, electrospray can offer a good atomization effect with excellent fine particle sizes. Wang et al. developed a matrix coating approach assisted by an electric field, which significantly improved the intensity of lipids, peptides, and proteins compared with vibration spray and sprayer gun and obtained good imaging spectra (Wang et al., 2015; Wang et al., 2016; Wang et al., 2017). Guo et al. developed an electric field-assisted scanning-spraying (EFASS) matrix coating system to deposit matrix on tissue with crystal sizes of less than 10 μm (Guo et al., 2015). Compared with airbrush and sublimation, the EFASS system could effectively enhance detection sensitivity and allow for uncovering more analytes in the tissue section.

**Figure 1:**
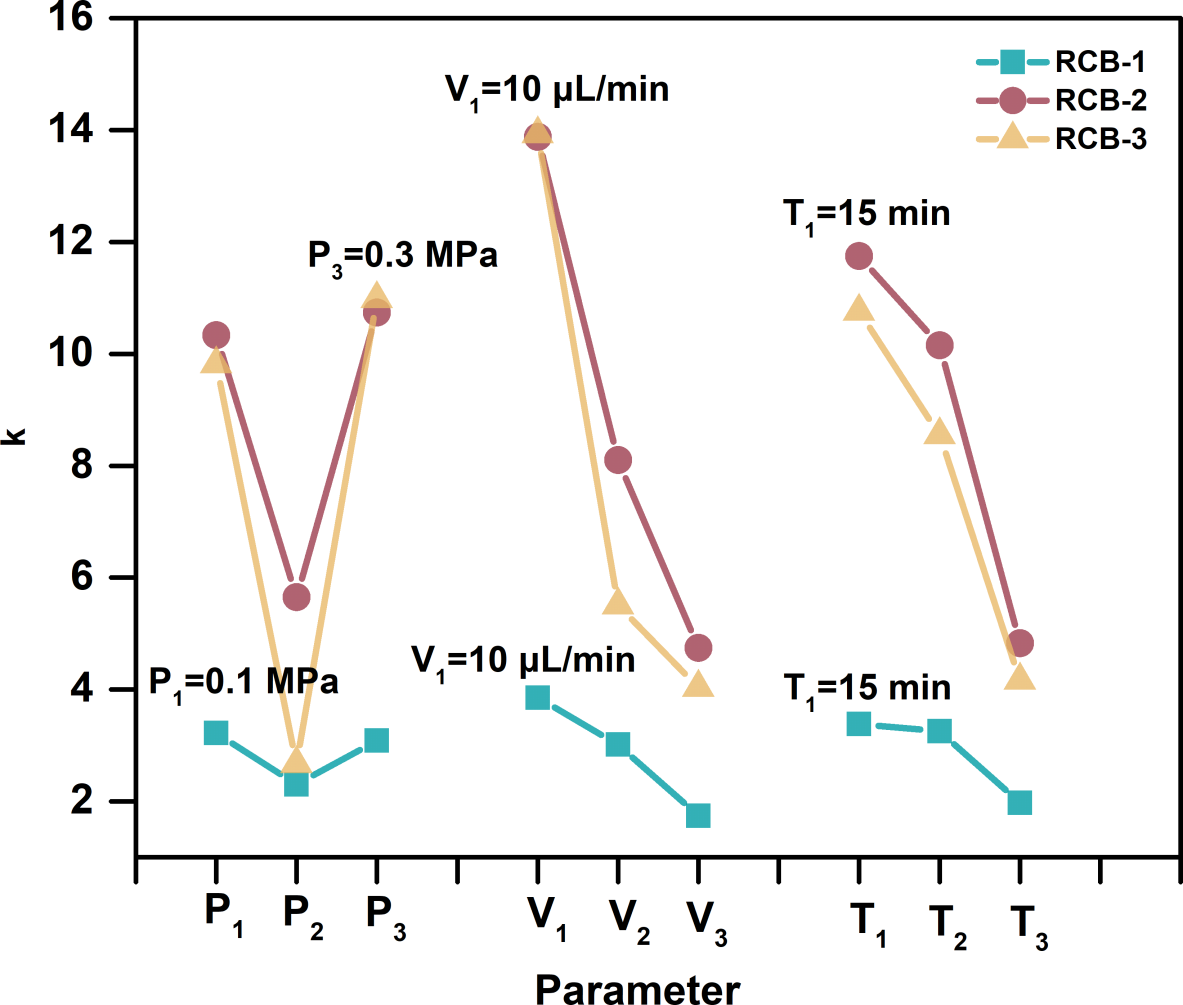
The optimization of the EFASS parameters by an orthogonal design of three factors and three levels. The Nitrogen pressure was 0.1 MPa, 0.2 MPa, and 0.3 MPa. The spraying velocity was 10 μL/min, 20 μL/min, and 30 μL/min, and the spraying time was 15 min, 30 min, and 45 min, respectively.

We employed EFASS to ensure a uniform matrix spraying effect in this work. Three parameters, the nitrogen pressure, the spraying velocity, and the spraying time, were optimized by an orthogonal design of three factors at three levels (**Figure 1**; **Table S4**). The primary and secondary dominants were spraying velocity and spraying time. The optimized parameters combination were the nitrogen pressure, 0.3 MPa, and the spraying velocity, 10 μL/min, and the spraying time, 15 min. Coating one tissue section of ca. 35 mm^2^ × 65 mm^2^ each time costs 150 μL of the matrix solution.

#### 3.1.4 Effect of washing protocol on MSI

The most delicate sample preparation step in MSI is tissue washing, which can directly alter the quantity of analytes in the sample and thus significantly change the spatial distribution. The current washing protocols are usually needed to be specifically designed based on the characteristics of the analyte and each type of tissue in MSI (Goodwin, 2012). According to the experimental knowledge from animal tissue sections ((Lemaire et al., 2006; Thomas et al., 2013), the organic solvents commonly used for washing are ethanol and isopropanol (Goodwin et al., 2008; van Hove et al., 2011). For example, Buchberger (Buchberger et al., 2020) et al. used seven different proportions of ethanol to water to optimize the washing protocol in crustacean brain tissue sections. The results showed that using 50% ethanol to wash sections for 10 s was the best washing condition. After washing, the intensity of 34 neuropeptides has been improved. Maslov et al. (Rešetar Maslov et al., 2019) systematically optimized 17 washing protocols for the MALDI-MSI analysis of peptides in dry-cured ham samples. When isopropanol was used for gradient elution, it effectively removed salt and fat and improved the intensity of peptides. In addition, it obviously improved the “sweet spot” effect of the matrix, increased the uniformity of the matrix, and obtained better MSI data.

To our knowledge, only one work reported the effect of organic solvents washing on MALDI-MSI analysis in plant tissues. Recently, Sun et al. significantly improved the sensitivity and coverage of metabolites in *Salvia miltiorrhiza Bge* by using acetone as a simple organic washing protocol in MALDI-MSI (Sun et al., 2020). The lipid removal effect of organic solvents washing on MALDI-MSI analysis of peptides in lipid-rich plant tissue sections has not been explored yet. Herein we investigated the washing protocol for defatting in detail for castor beans for the first time as a plant tissue material containing about 60% lipids.

In this paper, we selected and thoroughly discussed eight different washing protocols based on their known efficacy for lipid removal from the literature (Seeley et al., 2008; Deutskens et al., 2011; Yang and Caprioli, 2011; Rešetar Maslov et al., 2019) or with slight modification to reduce high-abundant lipid components and increase the intensity of peptide ions. Meanwhile, we introduced the Oil Red O staining method to visualize and monitor the lipid removal efficiency after the washing step. The dye Oil Red O is highly soluble in lipids and can specifically mark the lipid in the tissue as red or orange-red color. This staining method has already been commonly used in pathological diagnosis to display the lipid in the tissue but has not been applied to the MALDI-MSI technique.

**Figure 2:**
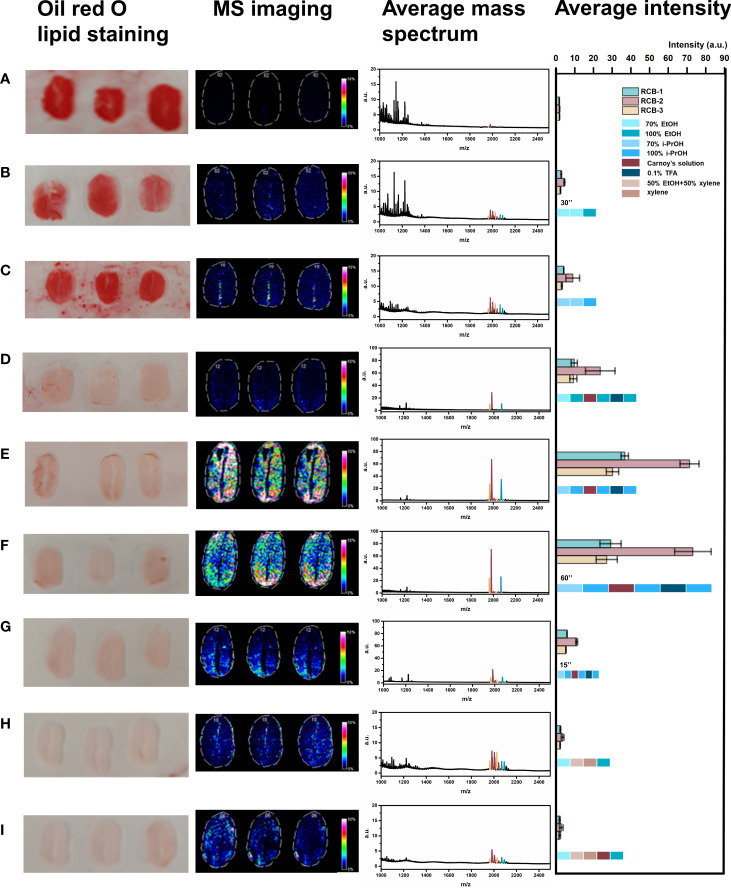
Comparison of different washing protocols. **(A)** No wash; **(B)** Wash with 70% ethanol, 70% ethanol, 100% ethanol for 30 s, respectively; **(C)** Wash with 70% isopropanol, 70% isopropanol, 100% isopropanol for 30 s, respectively; **(D)** Wash with 70% ethanol, 100% ethanol, Carnoy’s solution⃰, 100% ethanol, 0.1%TFA, 100% ethanol for 30 s, respectively; **(E)** Wash with 70% isopropanol, 100% isopropanol, Carnoy’s solution, 100% isopropanol, 0.1%TFA, 100% isopropanol for 30 s, respectively; **(F)** Wash with 70% isopropanol, 100% isopropanol, Carnoy’s solution, 100% isopropanol, 0.1%TFA, 100% isopropanol for 60 s, respectively; **(G)** Wash with 70% isopropanol, 100% isopropanol, Carnoy’s solution, 100% isopropanol, 0.1%TFA, 100% isopropanol for 15 s, respectively; **(H)** Wash with 70% ethanol, 1:1(v/v) mixture of ethanol and xylene, xylene, 100% ethanol for 30 s, respectively; **(I)** Wash with 70% ethanol, 1:1(v/v) mixture of ethanol and xylene, xylene, Carnoy’s solution, 100% ethanol for 30 s, respectively. (*Carnoy’s solution= 60% (v/v) ethanol, 30% (v/v) chloroform, 10% (v/v) glacial acetic acid).

**Figure 3:**
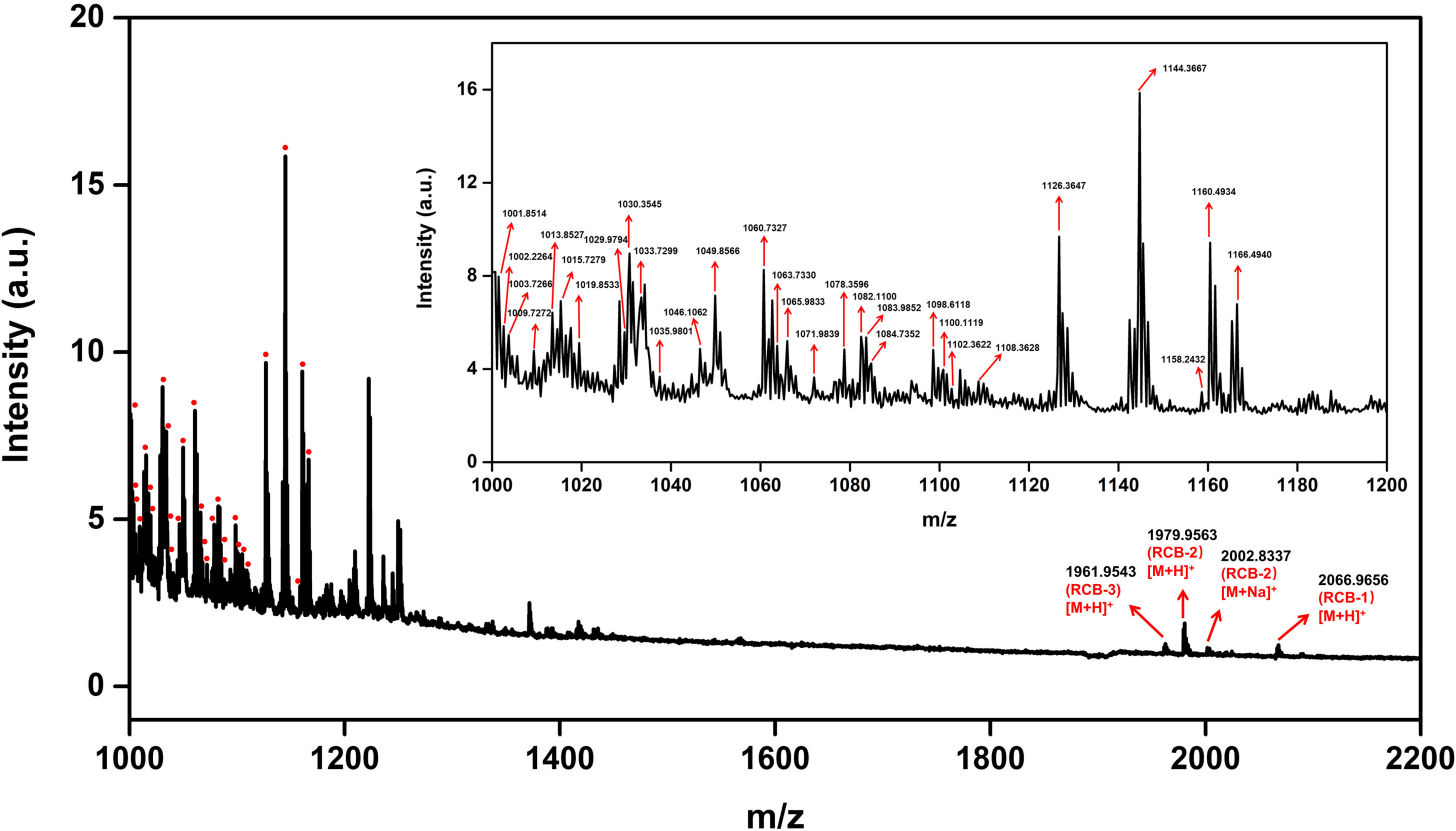
Average mass spectrum of tissue sections “no wash.” The red dot indicates the annotated lipid.

As shown in **Figure 2**, tissue sections were bright red in color for the “no wash” control (protocol A). In the average mass spectrum of MALDI-MSI, hundreds of peaks with high intensity in the range of m/z 1000~1200 were observed, while the intensity of RCBs in the range of m/z 1900~2100 was relatively low. We exported peaks in the range of m/z 1000~1200, up to 536 peaks, to LIPID MAPS database (https://lipidmaps.org) with a selection of fatty acyls, glycerolipids, glycerophospholipids, sphingolipids, sterol lipids ions ([M+H]^+^, [M+Na]^+^ and [M+K]^+^) for retrieval, and preliminarily matched 183 results, of which 30 peaks with strong intensity were marked in **Figure 3**. All the matching results were summarized in **Table S5** and the main matching results of 30 peaks were summarized in **Table 1**. These lipids may mainly be triacylglycerols, fatty esters, fatty acids, glycerophosphocholines, and phosphosphingolipids.

**Table 1:** LIPID MAPS database matching results.

Input Mass	Matched Mass	Delta	Name	Formula	Ion
1001.8514	1001.8532	0.0018	TG 63:8	C_66_H_112_O_6_	[M+H]^+^
1001.8514	1001.8508	0.0006	TG 61:5	C_64_H_114_O_6_Na	[M+Na]^+^
1002.2264	1002.2093	0.0171	CoA 11:2; O3	C_32_H_52_N_7_O_20_P_3_SNa	[M+Na]^+^
1002.2264	1002.2247	0.0017	CoA 12:1; O	C_33_H_56_N_7_O_18_P_3_SK	[M+K]^+^
1002.2264	1002.2611	0.0347	CoA 13:0	C_34_H_60_N_7_O_17_P_3_SK	[M+K]^+^
1003.7266	1003.7749	0.0483	TG 64:14	C_67_H_102_O_6_	[M+H]^+^
1003.7266	1003.7725	0.0459	TG 62:11	C_65_H_104_O_6_Na	[M+Na]^+^
1003.7266	1003.7151	0.0115	TG 61:12	C_64_H_100_O_6_K	[M+K]^+^
1009.7272	1009.7621	0.0349	TG 61:9	C_64_H_106_O_6_K	[M+K]^+^
1013.8527	1013.8532	0.0005	TG 64:9	C_67_H_112_O_6_	[M+H]^+^
1013.8527	1013.8508	0.0019	TG 62:6	C_65_H_114_O_6_Na	[M+Na]^+^
1013.8527	1013.8873	0.0346	TG 60:0	C_63_H_122_O_6_K	[M+K]^+^
1015.7279	1015.7725	0.0446	TG 63:12	C_66_H_104_O_6_Na	[M+Na]^+^
1015.7279	1015.7151	0.0128	TG 62:13	C_65_H_100_O_6_K	[M+K]^+^
1019.8533	1019.9001	0.0468	TG 64:6	C_67_H_118_O_6_	[M+H]^+^
1019.8533	1019.8977	0.0444	TG 62:3	C_65_H_120_O_6_Na	[M+Na]^+^
1019.8533	1019.8038	0.0495	TG 63:10	C_66_H_108_O_6_Na	[M+Na]^+^
1019.8533	1019.8403	0.0130	TG 61:4	C_64_H_116_O_6_K	[M+K]^+^
1029.9794	1029.9784	0.0010	TG 64:1	C_67_H_128_O_6_	[M+H]^+^
1030.3545	1030.3522	0.0023	CoA 18:2	C_39_H_66_N_7_O_17_P_3_S	[M+H]^+^
1030.3545	1030.3134	0.0411	CoA 15:0; O	C_36_H_64_N_7_O_18_P_3_SNa	[M+Na]^+^
1033.7299	1033.7621	0.0322	TG 63:11	C_66_H_106_O_6_K	[M+K]^+^
1035.9801	1035.9314	0.0487	TG 65:5	C_68_H_122_O_6_	[M+H]^+^
1046.1062	1046.0824	0.0238	FA 70:0; O2	C_70_H_140_O_4_	[M+H]^+^
1049.8566	1049.8508	0.0058	TG 65:9	C_68_H_114_O_6_Na	[M+Na]^+^
1049.8566	1049.8873	0.0307	TG 63:3	C_66_H_122_O_6_K	[M+K]^+^
1060.7327	1060.6907	0.0420	MIPC 40:0; O4	C_52_H_102_NO_18_P	[M+H]^+^
1063.7330	1063.7151	0.0179	TG 66:17	C_69_H_100_O_6_K	[M+K]^+^
1065.9833	1065.9760	0.0073	TG 65:1	C_68_H_130_O_6_Na	[M+Na]^+^
1071.9839	1072.0042	0.0203	CE 46:3; O2	C_73_H_130_O_4_	[M+H]^+^
1078.3596	1078.3522	0.0074	CoA 22:6	C_43_H_66_N_7_O_17_P_3_S	[M+H]^+^
1078.3596	1078.3497	0.0099	CoA 20:3	C_41_H_68_N_7_O_17_P_3_SNa	[M+Na]^+^
1078.7346	1078.7141	0.0205	MIPC 42:0; O2	C_54_H_106_NO_16_PNa	[M+Na]^+^
1082.1100	1082.0800	0.0300	FA 71:0; O2	C_71_H_142_O_4_Na	[M+Na]^+^
1083.9852	1083.9655	0.0197	TG 65:0	C_68_H_132_O_6_K	[M+K]^+^
1084.7352	1084.7635	0.0283	MIPC 44:0; O2	C_56_H_110_NO_16_P	[M+H]^+^
1084.7352	1084.7131	0.0221	PC 56:12	C_64_H_104_NO_8_PK	[M+K]^+^
1098.6118	1098.6466	0.0348	MIPC 40:0; O4	C_52_H_102_NO_18_PK	[M+K]^+^
1100.1119	1100.1294	0.0175	FA 74:1; O2	C_74_H_146_O_4_	[M+H]^+^
1102.3622	1102.4097	0.0475	CoA 22:2; O	C_43_H_74_N_7_O_18_P_3_S	[M+H]^+^
1102.3622	1102.3497	0.0125	CoA 22:5	C_43_H_68_N_7_O_17_P_3_SNa	[M+Na]^+^
1102.3622	1102.3499	0.0123	CoA 19:0; O	C_40_H_72_N_7_O_18_P_3_SK	[M+K]^+^
1108.3628	1108.3991	0.0363	CoA 24:5	C_45_H_72_N_7_O_17_P_3_S	[M+H]^+^
1108.3628	1108.3967	0.0339	CoA 22:2	C_43_H_74_N_7_O_17_P_3_SNa	[M+Na]^+^
1126.3647	1126.4073	0.0426	CoA 22:1; O	C_43_H_76_N_7_O_18_P_3_SNa	[M+Na]^+^
1126.3647	1126.3497	0.0150	CoA 24:7	C_45_H_68_N_7_O_17_P_3_SNa	[M+Na]^+^
1126.3647	1126.3863	0.0216	CoA 22:1	C_43_H_76_N_7_O_17_P_3_SK	[M+K]^+^
1144.3667	1144.3603	0.0064	CoA 24:6; O	C_45_H_70_N_7_O_18_P_3_SNa	[M+Na]^+^
1144.3667	1144.3969	0.0302	CoA 22:0; O	C_43_H_78_N_7_O_18_P_3_SK	[M+K]^+^
1144.3667	1144.3393	0.0274	CoA 24:6	C_45_H_70_N_7_O_17_P_3_SK	[M+K]^+^
1144.7417	1144.7846	0.0429	MIPC 46:0; O4	C_58_H_114_NO_18_P	[M+H]^+^
1158.2432	1158.2076	0.0356	FA 78:0; O2	C_78_H_156_O_4_	[M+H]^+^
1160.4934	1160.4879	0.0055	CoA 26:1; O	C_47_H_84_N_7_O_18_P_3_S	[M+H]^+^
1166.4940	1166.4749	0.0191	CoA 26:1	C_47_H_84_N_7_O_17_P_3_SNa	[M+Na]^+^

We then performed tissue washing with different organic solvents for salts and lipids removal (protocol B~I), including ethanol, isopropanol, chloroform, and xylene, in a stepwise way with a single kind or mixture form in different ratios. The commonly used organic solvent for removing salts and lipids was ethanol. For example, Yang et al. washed the tissue section first with 70% ethanol for 30 s and then with 100% ethanol for 30 s towards the rat brain tissue section (Yang and Caprioli, 2011). We evaluated this same washing protocol as protocol B. However, it did not significantly increase the intensity of RCBs while the red color was still maintained. In lieu of isopropanol in the same washing procedure provided a slight improvement in increasing the intensity of RCBs with a negligible defatting effect (protocol C).

Chloroform has a higher hydrophobility than ethanol and isopropanol, which was often used for lipid extraction. Carnoy’s solution, constituted by 60% *(v/v)* ethanol, 30% *(v/v)* chloroform, and 10% *(v/v)* glacial acetic acid, has been applied to the washing procedure to remove lipids in the MALDI-MSI experiment on dry-cured ham (Rešetar Maslov et al., 2019) and rat brain tissue section (Deutskens et al., 2011), and has achieved good effect. We also introduced this Carnoy’s solution to our protocol D. The tissue was firstly rinsed with 70% ethanol followed by 100% ethanol to fix the section, which is a standard step used in classical histology, then applied with Carnoy’s solution to remove most of the lipids from the section, followed by washing with 100% ethanol to remove the remaining chloroform from Carnoy’s solution. Next, 0.1% TFA aqueous solution removed salts, and a final step of 100% ethanol removed excess water. This washing protocol significantly increased the intensity of RCBs with a greatly reduced red color in a tissue section stained with Oil Red O. Besides, we found that Carnoy’s solution has a positive effect on maintaining the intact morphology of the fragile plant tissue sections in the process of tissue washing.

Similarly, ethanol was replaced with isopropanol in protocol E, which offered the most significant result in significantly reduced red color under Oil Red O staining. A high-quality RCBs distribution profile was shown in the images of MSI based on the average mass spectrum of strong RCBs signals. It indicated that, unlike animal tissue sections, isopropanol might be more conducive to lipid removal and peptide intensity enhancement in plant tissues than ethanol.

It was critical to strictly control the washing time in the process of tissue washing (Buchberger et al., 2020), and we further investigated the effect of washing duration on increasing the intensity of peptide in castor bean tissue sections based on the protocol E. When each washing step was lengthened to 60 s in protocol F, we found no significant difference in comparison with 30 s-interval (protocol E) but with a potential risk of delocalization of analytes. While each washing step only lasted 15 s (protocol G), the lipid removal was insufficient, as shown in darkening MSI images and decreased average intensity.

It has been reported that the alternative use of ethanol and xylene mixture achieved good dehydration and defatting effects in treating fat-rich tissues in pathology (Ding et al., 2019). Here we designed and tested another two protocols containing this kind of mixed reagents. Firstly, we consequentially applied 70% ethanol and 50% ethanol-50% xylene mixture to dehydrate the tissue sections of the castor bean. Because xylene is insoluble in water, it was used after dehydration treatment to dissolve the fat rapidly. Afterward, we added a single step of 100% ethanol (in protocol H) or further followed by Carnoy’s solution (in protocol I) to simultaneously amend the effect of dehydration and defatting, but neither protocol worked well. It suggested the introduction of xylene to defat the plant tissue section was not so ready and ideal. Finally, we determined that protocol E (Successively washing with 70% isopropanol, 100% isopropanol, Carnoy’s solution, 100% isopropanol, 0.1% TFA, and 100% isopropanol for 30 s, respectively) was the optimized condition for MALDI-MSI analysis of RCBs and other peptides.

In addition, we also roughly determined the recovery of RCBs by using the single point correction method to demonstrate the efficiency of the optimized washing protocol E. A mixed standard solution of 0.5 mg/L RCB-1, -2,-3 (containing 0.5 mg/L ^13^C_9_^15^N_1_-RCB-2 as IS) was prepared to calculate the concentration of all samples. Towards adjacent tissue sections from Xinjiang, in which three consecutive tissue sections in parallel on one ITO slide, the consecutive washing solutions in six steps were separately collected and concentrated to 1 mL, and then 0.5 mg/L ^13^C_9_^15^N_1_-RCB-2 were added as IS for MALDI-MS determination. Considering that the single tissue section area from Xinjiang is about 50 mm^2^, the thickness is 20 μm, and the density is regarded as 1 mg/mm^3^, therefore, its weight is 1 mg. The recovery of RCBs should be calculated as the lost content divided by the total content of RCBs in 1 mg tissue.

For the estimation of total content of RCBs in 1 mg tissue, we treated 100 mg castor bean homogenate with 1 mL of 50% ACN:0.1% TFA aqueous solution to avoid content derivation from a single tissue section and would adopt an average value in the calculation (detailed in Supplementary experimental). The results showed that RCBs were only slightly lost in step five (0.1% TFA washing) and step six (100% isopropanol washing). The recoveries of RCB-1 to 3 were 95.9%, 88.2%, and 90.3%, respectively (**Figure S2**). It indicated that the current optimized washing protocol E can maintain and expose the largest amount of target RCB peptides toward castor bean tissues.

Meanwhile, since that it is hard to observe RCBs without tissue washing, we can not directly judge the components drift by comparing the distribution before and after washing. We regard that this tissue washing protocol may not cause components drift, or the range of drift is very small. First, according to the reports in the literature, all three RCBs contain two pairs of disulfide bonds, the structure made them stable in the surrounding microenvironment. Second, we did not detect RCBs on ITO slides around the cleaned tissue sections. Third, we have carried out a preliminary *in situ* digestion experiment of proteins on castor beans tissue sections, and found that after tissue section washing by protocol E, enzymatic incubation, matrix coating and other steps, high abundance of RCBs can still be detected in the tissue section, which also indicated that RCBs can stably exist in the tissue.

### 3.2 Distribution of RCBs in castor beans

In previous work, we have performed *de novo* sequencing to verify the sequences of high disulfide bonds abundant RCBs by MALDI-MS/MS (**Supplementary Data**; **Figure S3**) (He et al., 2021). The sequence characteristics of RCBs are very similar to that of a class of plant peptides called “plant defensins” reported in the literature (Thevissen et al., 2007). Therefore, RCBs may be related to their own detoxification. In addition, in the identification and detection view, peptides are better candidates offering better sensitivity and easier pretreatment due to their simpler structures than ricin.

**Figure 4:**
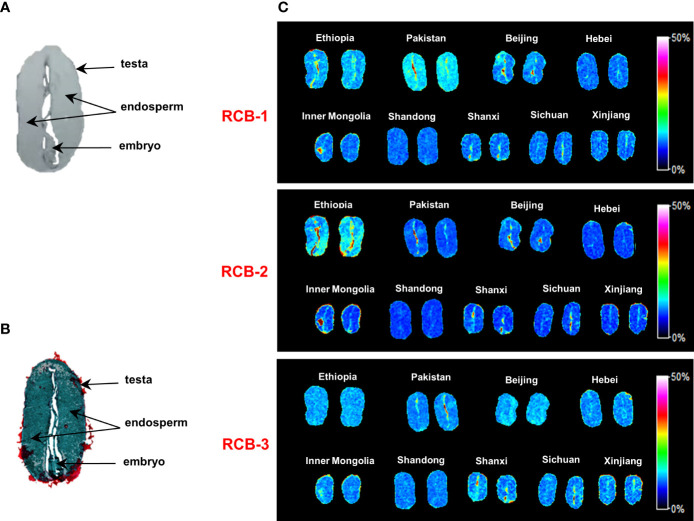
MALDI-MSI analysis of RCBs in positive ion mode in castor bean sections. **(A)** Optical imaging of the castor bean structure; **(B)** Saffron O-fast green plant tissue staining; **(C)** Distribution of RCB-1, -2 and -3 in castor beans from geographical sources. Color scales encode arbitrary ion relative strength.

**Figures 4A, B** showed the optical image and Saffron O-fast histological staining image of medial longitudinal castor bean sections with arrows indicating endosperm, the embryo (embryonic axis and cotyledons), and testa tissues. To investigate the distribution of RCBs in castor beans, we performed MALDI-MSI analysis and obtained MS images with a spatial resolution of 100 μm. **Figure 4C** was MS imaging on castor beans from night geographical locations. RCB-1 to -3 were generally distributed in the whole endosperm but not uniform and mainly enriched in testa and embryo tissue in some geographical locations, especially in Ethiopia and Pakistan. Our results show that RCB-1 to 3 were compartmentalized differently among the embryo and testa tissues from different countries (Ethiopia, Pakistan, and China). For all castor bean sections from nine geographic origins, we did not find either RCB-4 or RCB- as Ovenden et al. reported towards the *“impala”* cultivar from Tanzania (Ovenden et al., 2014).

### 3.3 Quantitative determination of RCBs in castor beans from different geographical origins

**Figure 5:**
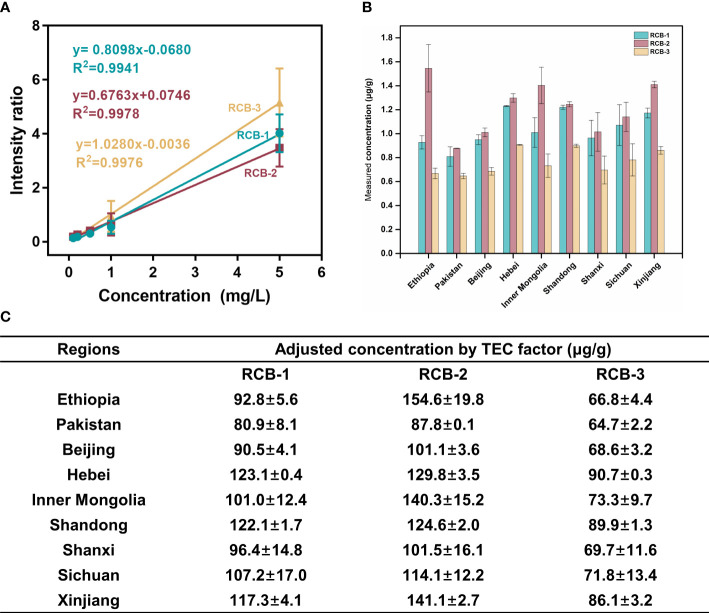
Quantitation of RCB-1,-2 and -3 in castor bean tissues from nine geographical sources by MALDI-MSI. **(A)** Calibration curves of RCB-1, -2, and -3, the error bars in the graph represented the standard deviation of three microspots for each calibration solution; **(B)** The measured concentrations, and **(C)** the adjusted concentrations by TEC factor of RCB-1, -2, and -3 in castor bean tissues from nine geographical sources.

Due to the tissue heterogeneity, matrix coating heterogeneity, poor analyte extraction, and ionization suppression effects, the quantitative analysis of MALDI-MSI is still challenging. Currently, SILIS has been reported to calibrate the variability of ion suppression to maximize quantitative reproducibility, and it is the most pervasive method in MSI (Pirman et al., 2013; Havlikova et al., 2019). Herein, we also used SILIS to establish the standard curve and calculate the content of RCBs in tissue sections. The calibration curves generated by plotting the average intensity ratio of RCB-1, RCB-2, and RCB-3 to the ^13^C_9_^15^N_1_-RCB-2 versus the concentration of RCB-1 to -3 applied to the ITO slide were shown in **Figure 5A**. RCB-1, RCB-2, and RCB-3 all had good linearity (R^2 =^ 0.99). The standard peptide solution was directly spotted on the ITO slide to generate standard curves, which did not consider the matrix effect of the tissue itself.

The average intensity ratio (RCB-1,2,3/^13^C_9_^15^N_1_-RCB-2) of castor bean tissue sections was introduced to the standard curve equation to calculate the content of RCBs in tissue sections. To obtain the quantitative concentration in gram per gram of tissue, the density of castor bean tissue is assumed to be 1 mg/mm^3^. The volume in mm^3^ was obtained by multiplying the area value of the tissue section by the thickness of the section. The final concentration of RCBs in g/g of tissue was obtained by comparing the calculated weight of RCBs with the total weight of the tissue. The quantitative data are summarized in **Fiugre 5**. Three RCB peptides were present in varying amounts in nine different geographic sources, the content of RCB-2 was the highest, followed by RCB-1, and the content of RCB-3 was the lowest in any source. It was similar to the content of RCB-1 to -3 in other cultivars of castor beans reported in the literature, that is, RCB-1 and RCB-2 existed in higher amounts in all cultivars, while RCB-3 was present only in some cultivars (Ovenden et al., 2009). Although the distribution of tissue sections was different, the content of RCBs in nine geographical sources was roughly the same.

It should be noted that the direct measured RCB content in the tissue section *via* this SILIS way was order of magnitude lower than the determined value towards averaged castor bean homogenate. Possible reason lies in that, the standard curve made on ITO slide surface is quite different from which on the tissue, and the endogenous components in the tissue section hinder the ionization of target RCBs and IS peptides. Hereby we introduced the tissue extinction coefficient (TEC) to amend the ion suppression of RCBs in the tissue section and resolve the discrepancy problem. The TEC of the tissue section was *ca.* 0.01 (detailed in **Supplementary experimental**; **Table S6**), so the adjusted RCB content was calculated from the direct measured RCB content divided by the TEC factor, and it can approach the measured value in homogenates, indicating an effective elimination of tissue matrix effect.

### 3.4 Multivariate statistical analysis of castor beans from different geographical origins

**Figure 6:**
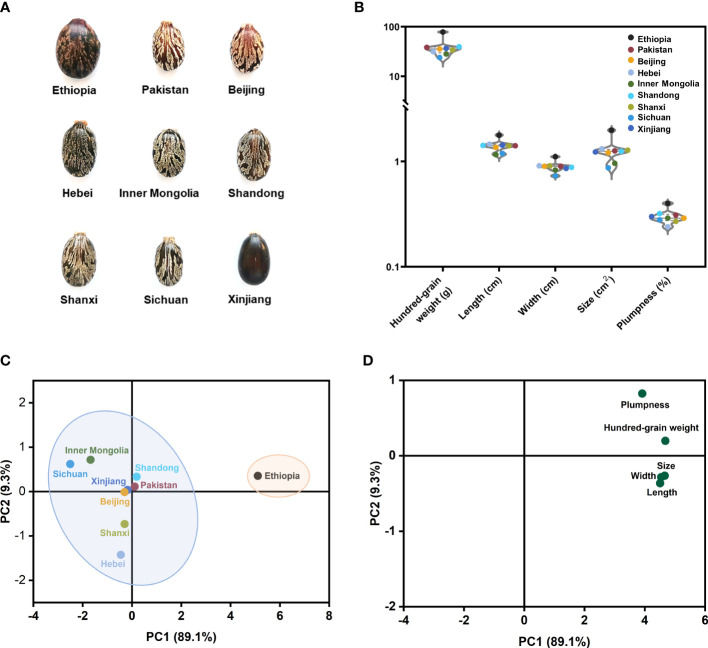
Phenotype description and PCA analysis of castor beans from nine different sources. **(A)** Appearance and morphology of castor beans; **(B)** Violin plot of five phenotypic traits of castor beans. Score plot **(C)** and loading plot **(D)** of the PCA analysis model based on five features of castor beans from different geographical sources.

Regarding the castor beans from nine different geographical sources with five important phenotype traits, we found no significant difference except for castor beans from Ethiopia, which had relatively large sizes and weights with a distinctive appearance (**Figure 6A**). Considering that PCA recombines the original variables into a group of new comprehensive variables that are independent of each other, ideally, samples with similar compound types and amounts will cluster together in the scores plot of the PCA model, PCA with five traits as characteristic values was used to explore whether it is possible to distinguish castor beans of nine different sources only from their appearance (**Figure 6**). From the score plot of the principal component (PC1) and PC2, PC1 explained 89.1%, and PC2 explained 9.3% of the total data variation and the high values of R^2^ and Q^2^ manifest the outstanding prediction capability of the PCA models and well explain the cumulative variation of the PCs of the data (R^2 =^ 0.983, Q^2 =^ 0.914). The geographical origins of castor beans from Ethiopia were clustered into one category, and others (seven China sources and Pakistan) were clustered into another category. It indicated that the five phenotype traits of castor beans from other eight geographical sources were similar except for Ethiopia, therefore, castor beans can hardly be distinguished from each other only in terms of their appearance traits. From the loading plot, no significant difference in the contribution of the five characters to the PC1, indicating that hundred-grain weight, length, width, size and plumpness made almost no difference a considerable contribution to the classification of Ethiopia and other eight sources. We hope to explore further the original traceability of the castor beans at the molecular level *via* the MSI technique.

**Figure 7:**
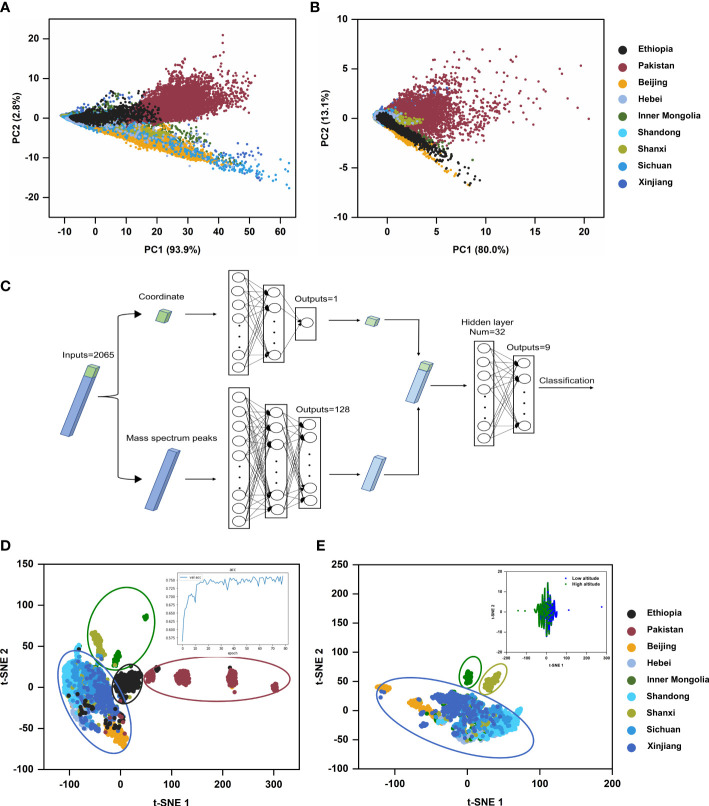
Multivariate statistical analysis results of castor beans from different geographical sources. **(A)** Score plot of the MSI dataset of all mass spectra peaks in PCA model; **(B)** Score plot of the MSI dataset of the mass spectra peaks of RCBs in PCA model; **(C)** Schematic diagram of DNN model; **(D)** t-SNE visual scatter plot of DNN model based on MSI dataset from nine geographical sources, the inset was the accuracy rate curve of neural network; **(E)** t-SNE plot of DNN model based on MSI dataset from seven geographical sources in China, the inset was a t-SNE plot of DNN model based on MSI dataset from different altitudes.

Hence, we employed PCA as an unsupervised analysis model to perform the dimension reduction of MSI data of castor bean samples from nine geographical regions, including data sets of all peaks and data sets containing only RCBs, using protocol E in the sample pretreatment. Data sets of all peaks were generated automatically with the TIC normalization by “Find peaks” tool and data sets containing only RCBs was generated by manual m/z selection on SCiLS Lab 2016b. The Peak Alignment tool was then used to align all peaks. **Figure 7** shows the scores plot of the two PCs for the regional distribution of castor beans. For the data sets of all peaks, the score plot from PC1 (93.9%) and PC2 (2.8%) accounted for 96.7% of the total variance observed across the groups. Despite some overlapping profiles in nine groups, there was a separate trend among the different countries (Ethiopia, Pakistan and China), indicating the major distinct feature existed among different countries. Provinces and autonomous regions in China were almost clustered into one category, indicating that the type and content of chemical components in China were somewhat common. Similar results could also be obtained from PCA analysis of data sets containing mere RCBs (**Figure 7B**). The score plot from PC1 (80.0%) and PC2 (13.1%) accounted for 93.1% of the total variance. It suggested that the RCBs as an index have some unique region attribution value.

MSI experiment obtained a three-dimensional data cube with spatial coordinates (x and y) and an m/z axis containing the spectrum information. However, the available spatial information was lost and abandoned in most data analysis of MSI. The advent of deep learning, a class of models and associated learning methods based on artificial neural networks, has greatly facilitated such spatial analysis and may offer a promising strategy for classifying MSI data as it has been successfully applied to image classification. For example, Behrmann et al. proposed an adapted architecture based on deep convolutional networks to classify two lung tumor subtypes (Behrmann et al., 2018). DNN is a robust neural network, in its composition of multiple network layers, the input layer(s), hidden layer(s), and output layer(s) are set in different positions. Each layer is fully connected, and the output from each layer is an input to the next. The unique characteristic of DNN is, after feature mapping layer by layer, DNN maps the features of the existing spatial samples to another space to learn better feature expression for the current input. Here we firstly examined the classification potential of the dual-channel DNN. This DNN was designed according to the fabrication feature of MSI data, *i.e.*, each sample data was defined by two types of characteristics, the position of the sample point (the coordinate) and mass spectra information in this point (**Figure 7C**).

For both channels, two types of different features of the sample data were separately input into two sub-networks and then simultaneously trained. The features extracted from the two sub-networks were concatenated and inputted into the third sub-network with two fully connected layers for classification. After five repeated experiments, the average classification accuracy of the dual-channel DNN model for nine samples from different geographical sources was beyond 76%. Afterward, we used t-SNE to reduce the 32-dimensional features extracted from the first layer in the third sub-networks into a two-dimensional scatter plot, which distinctly displayed the distribution characteristics of samples from different geographical sources.

DNN provided more insights in comparison with PCA analysis. (i) It indicated a distinct trend among three countries, Ethiopia, Pakistan, and China. (ii) Inner Mongolia, Shanxi, and the other five provinces or autonomous regions were clustered into three groups among all seven regions of China (**Figures 7D, E**), disclosing a new clue from the viewpoint of altitude difference. Inner Mongolia and Shanxi are located on the Mongolian plateau, and the other five provinces or autonomous regions are located in flat or basin areas on China mainland. We also inputted the dichotomous sample data composed of different altitudes into the dual-channel DNN model for training, and the accuracy of the test set reached 85%. (iii) Furthermore, the feature importance analysis using SHAP for nine respective sources (**Figure S4**) revealed that among the top 20 features with SHAP values among the 2065-dimensional features, the position (x, y) in the tissue section always has the prior rank no matter which geographic origin. It indicated the importance of analyzing the integrated MSI data as a whole set since the peak intensity values of mass spectra of sample points in the same tissue section from different geographical sources differed.

## 4 Conclusions

In this work, we developed a comprehensive tissue pretreatment protocol for MALDI-MSI analysis of endogenous peptide molecules in the castor bean containing plenty of lipids. In particular, we discovered a modified six-step organic solvents washing protocol containing alternative rinses of isopropanol and Carnoy’s solution to provide a good lipid removal effect as well aa s significant improvement on the sensitivity of endogenous peptide biomarkers, RCBs, in castor bean tissues. It is the first report to depict good images of peptides inside lipid-rich plant tissue. With the other optimized crucial steps in sample preparation, we ensured the construction of a robust MALDI-MSI method to enhance the detection sensitivity of RCBs in castor beans. We then clearly mapped the spatial distribution and quantified RCBs in castor bean tissue sections, as well as from nine different geographic sources from seven regions of China and two regions from Ethiopia and Pakistan. Employed with multivariate statistical models of PCA, DNN, and t-SNE, the interpretation of the generated MSI data revealed valuable classification clues from nationality and altitude, in which RCBs contribute specific values as geographic attribution biomarkers. It put forward a task of further examination work on more geographical origins of castor bean plants. This method provides a new research perspective for the traceability of castor bean-relevant intoxication events.

## Data availability statement

The original contributions presented in the study are included in the article/**Supplementary Material**. Further inquiries can be directed to the corresponding authors.

## Author contributions

LQ conducted the experiments, analyzed data, and interpreted the results. LQ wrote the primary manuscript with support from BX. JH assisted in the multivariate statistical analysis of the DNN and wrote the relevant content with support from SH. CW assisted in this experiment. DT provided castor beans from Ethiopia, Pakistan, and Shandong province and a full discussion of the relevant investigation. LG supervised the experiment, revised the manuscript, and provided financial support, XB and JX reviewed the manuscript and approved it for the final release. All authors provided critical feedback, helped shape the research, and authorized the final manuscript.

## References

[B1] Aggarwal R. Aggarwal H. Chugh P. (2017). Medical management of ricin poisoning. J. Med. Allied. Sci. 7, 82. doi: 10.5455/jmas.259532

[B2] Behrmann J. Etmann C. Boskamp T. Casadonte R. Kriegsmann J. Maass P. (2018). Deep learning for tumor classification in imaging mass spectrometry. Bioinformatics 34, 1215–1223. doi: 10.1093/bioinformatics/btx724 29126286

[B3] Bencivenni M. Faccini A. Zecchi R. Boscaro F. Moneti G. Dossena A.. (2014). Electrospray MS and MALDI imaging show that non-specific lipid-transfer proteins (LTPs) in tomato are present as several isoforms and are concentrated in seeds. J. Mass Spectrom. 49, 1264–1271. doi: 10.1002/jms.3454 25476944

[B4] Bezerra M. A. Santelli R. E. Oliveira E. P. Villar L. S. Escaleira L. A. (2008). Response surface methodology (RSM) as a tool for optimization in analytical chemistry. Talanta 76, 965–977. doi: 10.1016/j.talanta.2008.05.019 18761143

[B5] Blutke A. Sun N. Xu Z. H. Buck A. Harrison L. Schriever S. C.. (2020). Light sheet fluorescence microscopy guided MALDI-imaging mass spectrometry of cleared tissue samples. Sci. Rep. 10, 13. doi: 10.1038/s41598-020-71465-1 32879402 PMC7468256

[B6] Buchberger A. R. Vu N. Q. Johnson J. Delaney K. Li L. (2020). A simple and effective sample preparation strategy for MALDI-MS imaging of neuropeptide changes in the crustacean brain due to hypoxia and hypercapnia stress. J. Am. Soc Mass Spectrom. 31, 1058–1065. doi: 10.1021/jasms.9b00107 32150406 PMC7467133

[B7] Darby S. M. Miller M. L. Allen R. O. (2001). Forensic determination of ricin and the alkaloid marker ricinine from castor bean extracts. J. Forensic Sci. 46, 1033–1042. doi: 10.1520/JFS15097J 11569541

[B8] Deutskens F. Yang J. Caprioli R. M. (2011). High spatial resolution imaging mass spectrometry and classical histology on a single tissue section. J. Mass Spectrom. 46, 568–571. doi: 10.1002/jms.1926 21630385 PMC3118566

[B9] Ding W. Zhou L. Tang W. Ying L. Yao H. (2019). Introduction of a method for removing fat during tissue dehydration. Chin. J. Pathol. 48, 248–250. doi: 10.3760/cma.j.issn.0529-5807.2019.03.017 30831656

[B10] Duriez E. Fenaille F. Tabet J. C. Lamourette P. Hilaire D. Becher F.. (2008). Detection of ricin in complex samples by immunocapture and matrix-assisted laser desorption/ionization time-of-flight mass spectrometry. J. Proteome Res. 7, 4154–4163. doi: 10.1021/pr8003437 18651759

[B11] Fredriksson S. Wunschel D. S. Lindström S. W. Nilsson C. Wahl K. Åstot C. (2018). A ricin forensic profiling approach based on a complex set of biomarkers. Talanta 168, 628–635. doi: 10.1016/j.talanta.2018.03.070 29784413

[B12] Gemperline E. Keller C. Jayaraman D. Maeda J. Sussman M. R. Ané J. M.. (2016). Examination of endogenous peptides in medicago truncatula using mass spectrometry imaging. J. Proteome Res. 15, 4403–4411. doi: 10.1021/acs.jproteome.6b00471 27726374 PMC6314849

[B13] Goodwin R. J. A. (2012). Sample preparation for mass spectrometry imaging: small mistakes can lead to big consequences. J. Proteomics 75, 4893–4911. doi: 10.1016/j.jprot.2012.04.012 22554910

[B14] Goodwin R. J. Pennington S. R. Pitt A. R. (2008). Protein and peptides in pictures: imaging with MALDI mass spectrometry. Proteomics 8, 3785–3800. doi: 10.1002/pmic.200800320 18712772

[B15] Greco V. Piras C. Pieroni L. Ronci M. Putignani L. Roncada P.. (2018). Applications of MALDI-TOF mass spectrometry in clinical proteomics. Expert Rev. Proteomics 15, 683–696. doi: 10.1080/14789450.2018.1505510 30058389

[B16] Guo S. Wang Y. Zhou D. Li Z. (2015). Electric field-assisted matrix coating method enhances the detection of small molecule metabolites for mass spectrometry imaging. Anal. Chem. 87, 5860–5865. doi: 10.1021/ac504761t 26016507

[B17] Havlikova J. Randall E. C. Griffiths R. L. Swales J. G. Goodwin R. J. A. Bunch J.. (2019). Quantitative imaging of proteins in tissue by stable isotope labeled mimetic liquid extraction surface analysis mass spectrometry. Anal. Chem. 91, 14198–14202. doi: 10.1021/acs.analchem.9b04148 31660728 PMC7007001

[B18] He W. Wang C. Yang J. Xu B. Guo L. Xie J. (2021). Tracing origins of castor beans through potential defensin peptides unraveled by MALDI mass spectrometry imaging. FENXI CESHI XUEBAO (J. Instrum. Anal.) 40, 543–550. doi: 10.3969/j.issn.1004-4957.2021.04.015

[B19] Keller C. Gemperline E. Li L. (2020). MALDI mass spectrometry imaging of peptides in medicago truncatula root nodules. Methods Mol. Biol. 2139, 341–351. doi: 10.1007/978-1-0716-0528-8_25 32462598 PMC7430052

[B20] Lemaire R. Wisztorski M. Desmons A. Tabet J. C. Day R. Salzet M.. (2006). MALDI-MS direct tissue analysis of proteins: Improving signal sensitivity using organic treatments. Anal. Chem. 78, 7145–7153. doi: 10.1021/ac060565z 17037914

[B21] Li B. Zhang Y. Ge J. Liu K. Li P. (2018). Sample preparation for mass spectrometry imaging of leaf tissues: a case study on analyte delocalization. Anal. Bioanal. Chem. 410, 7449–7456. doi: 10.1007/s00216-018-1355-5 30215125

[B22] Neagu A. N. (2019). Proteome Imaging: From Classic to Modern Mass Spectrometry-Based Molecular Histology. Adv. Exp. Med. Biol. 1140, 55–98. doi: 10.1007/978-3-030-15950-4_4 31347042

[B23] Ovenden S. P. B. Fredriksson S. A. Bagas C. K. Bergström T. Thomson S. A. Nilsson C.. (2009). *De novo* sequencing of RCB-1 to -3: peptide biomarkers from the castor bean plant ricinus communis. Anal. Chem. 81, 3986–3996. doi: 10.1021/ac900371y 19391602

[B24] Ovenden S. P. Pigott E. J. Rochfort S. Bourne D. J. (2014). Liquid chromatography-mass spectrometry and chemometric analysis of ricinus communis extracts for cultivar identification. Phytochem. Anal. 25, 476–484. doi: 10.1002/pca.2519 24737411

[B25] Pigott E. J. Roberts W. Ovenden S. P. B. Rochfort S. Bourne D. J. (2011). Metabolomic investigations of ricinus communis for cultivar and provenance determination. Metabolomics 8, 634–642. doi: 10.1007/s11306-011-0355-7

[B26] Pirman D. A. Reich R. F. Kiss A. Heeren R. M. Yost R. A. (2013). Quantitative MALDI tandem mass spectrometric imaging of cocaine from brain tissue with a deuterated internal standard. Anal. Chem. 85, 1081–1089. doi: 10.1021/ac302960j 23214490

[B27] Polito L. Bortolotti M. Battelli M. G. Calafato G. Bolognesi A. (2019). Ricin: an ancient story for a timeless plant toxin. Toxins (Basel) 11, 324. doi: 10.3390/toxins11060324 31174319 PMC6628454

[B28] Rešetar Maslov D. Svirkova A. Allmaier G. Marchetti-Deschamann M. Kraljević Pavelić S. (2019). Optimization of MALDI-TOF mass spectrometry imaging for the visualization and comparison of peptide distributions in dry-cured ham muscle fibers. Food Chem. 283, 275–286. doi: 10.1016/j.foodchem.2018.12.126 30722871

[B29] Seeley E. H. Oppenheimer S. R. Mi D. Chaurand P. Caprioli R. M. (2008). Enhancement of protein sensitivity for MALDI imaging mass spectrometry after chemical treatment of tissue sections. J. Am. Soc Mass Spectrom. 19, 1069–1077. doi: 10.1016/j.jasms.2008.03.016 18472274 PMC2582528

[B30] Sturtevant D. Romsdahl T. B. Yu X. H. Burks D. J. Azad R. K. Shanklin J.. (2019). Tissue-specific differences in metabolites and transcripts contribute to the heterogeneity of ricinoleic acid accumulation in Ricinus communis L. (castor) seeds. Metabolomics 15, 6. doi: 10.1007/s11306-018-1464-3 30830477

[B31] Sun C. Liu W. Ma S. Zhang M. Geng Y. Wang X. (2020). Development of a high-coverage matrix-assisted laser desorption/ionization mass spectrometry imaging method for visualizing the spatial dynamics of functional metabolites in salvia miltiorrhiza bge. J. Chromatogr. A 1614, 460704. doi: 10.1016/j.chroma.2019.460704 31753480

[B32] Tang X. Zhao M. Chen Z. Huang J. Chen Y. Wang F.. (2021). Visualizing the spatial distribution of metabolites in clausena lansium (Lour.) skeels using matrix-assisted laser desorption/ionization mass spectrometry imaging. Phytochemistry 192, 112930. doi: 10.1016/j.phytochem.2021.112930 34481177

[B33] Thevissen K. Kristensen H. H. Thomma B. P. Cammue B. P. François I. E. (2007). Therapeutic potential of antifungal plant and insect defensins. Drug Discovery Today 12, 966–971. doi: 10.1016/j.drudis.2007.07.016 17993416

[B34] Thomas A. Patterson N. H. Laveaux Charbonneau J. Chaurand P. (2013). Orthogonal organic and aqueous-based washes of tissue sections to enhance protein sensitivity by MALDI imaging mass spectrometry. J. Mass. Spectrom. 48, 42–48. doi: 10.1002/jms.3114 23303746

[B35] Van Hove E. R. Smith D. F. Fornai L. Glunde K. Heeren R. M. (2011). An alternative paper based tissue washing method for mass spectrometry imaging: localized washing and fragile tissue analysis. J. Am. Soc Mass. Spectrom. 22, 1885–1890. doi: 10.1007/s13361-011-0203-z 21952901 PMC3177040

[B36] Vu N. Q. Buchberger A. R. Johnson J. Li L. (2021). Complementary neuropeptide detection in crustacean brain by mass spectrometry imaging using formalin and alternative aqueous tissue washes. Anal. Bioanal. Chem. 413, 2665–2673. doi: 10.1007/s00216-020-03073-x 33403426 PMC8012219

[B37] Vuckovic I. Rapinoja M. L. Vaismaa M. Vanninen P. Koskela H. (2016). Application of comprehensive NMR-based analysis strategy in annotation, isolation and structure elucidation of low molecular weight metabolites of ricinus communis seeds. Phytochem. Anal. 27, 64–72. doi: 10.1002/pca.2600 26464348

[B38] Wang X. Han J. Hardie D. B. Yang J. Borchers C. H. (2016). The use of matrix coating assisted by an electric field (MCAEF) to enhance mass spectrometric imaging of human prostate cancer biomarkers. J. Mass. Spectrom. 51, 86–95. doi: 10.1002/jms.3728 26757076

[B39] Wang X. Han J. Hardie D. B. Yang J. Pan J. Borchers C. H. (2017). Metabolomic profiling of prostate cancer by matrix assisted laser desorption/ionization-fourier transform ion cyclotron resonance mass spectrometry imaging using matrix coating assisted by an electric field (MCAEF). Biochim. Biophys. Acta Proteins Proteomics 1865, 755–767. doi: 10.1016/j.bbapap.2016.12.012 28017863

[B40] Wang X. Han J. Yang J. Pan J. Borchers C. H. (2015). Matrix coating assisted by an electric field (MCAEF) for enhanced tissue imaging by MALDI-MS. Chem. Sci. 6, 729–738. doi: 10.1039/c4sc01850h 28706636 PMC5494562

[B41] Worbs S. Köhler K. Pauly D. Avondet M. A. Schaer M. Dorner M. B.. (2011). Ricinus communis intoxications in human and veterinary medicine-a summary of real cases. Toxins (Basel) 3, 1332–1372. doi: 10.3390/toxins3101332 22069699 PMC3210461

[B42] Yang J. Caprioli R. M. (2011). Matrix sublimation/recrystallization for imaging proteins by mass spectrometry at high spatial resolution. Anal. Chem. 83, 5728–5734. doi: 10.1021/ac200998a 21639088 PMC3136623

