# Supplementary material for: Key defatting tissue pretreatment protocol for enhanced MALDI MS Imaging of peptide biomarkers visualization in the castor beans and their attribution applications
Source: Front Plant Sci. 2022 Dec 16;13:1083901. doi: 10.3389/fpls.2022.1083901 (PMC9800866; doi:10.3389/fpls.2022.1083901)
Supplement: Supplementary file 1 [file DataSheet_1.pdf]

Supplementary Material

1 Supplementary experimental

***De novo* sequencing.** *De novo* sequencing by MALDI-MS/MS was performed to determine the sequences of RCB-1 to 3 in tissue section. To reduce the disulfide bridges to free Cys, a final concentration of 0.1 mol/L dithiothreitol was added to the matrix of 7 g/L CHCA and formed as a new matrix for MSI. After incubated at 37 °C for 30 minutes, both the group with dithiothreitol in the matrix and the control without dithiothreitol were analyzed under reflection positive mode in a LIFT fragmentation (Autoflex III MALDI TOF/TOF MS, Bruker Daltonik, Germany). The collected data were transferred to and analyzed by *de novo* sequencing in Biotools software (Bruker Daltonik, Germany), then in comparison with the reference sequence constructed by Sequence Editor software (Bruker Daltonik, Germany).

Recovery of RCBs after washing procedures. The recovery of RCBs in the tissue section after washing was determined by using the single point correction method. A mixed standard solution of 0.5 mg/L RCB-1,-2,-3 (containing 0.5 mg/L $^{13}C_9{}^{15}N_1$-RCB-2 as IS) was prepared to calculate the concentration of all samples. Towards three consecutive tissue sections from Xinjiang in parallel on one ITO slide, the consecutive washing solutions in six steps of washing protocol E were separatedly collected and concentrated to 1 mL, and then 0.5 mg/L $^{13}C_9{}^{15}N_1$-RCB-2 were added as IS for MALDI-MS determination. The samples were named S1, S2, S3, S4, S5, and S6, respectively. All sample was diluted 1.25 times and then determined.

The weight of a single tissue section was estimated from the tissue section area, the thickness, and the density. The single tissue section area from Xinjiang was about 50 mm^2, the thickness was 20 μm, and the density was regarded as 1 mg/mm^3, therefore, its weight was 1 mg. The recovery of RCBs should be calculated as the lost content divided by the total content of RCBs in 1 mg tissue.

For the estimation of total content of RCBs in 1 mg tissue, 100 mg castor bean homogenate were treated to avoid of content derivation from a single tissue section and an average value would be adopted in the calculation. The castor beans from Xinjiang were peeled and weighed 100 mg, then homogenized with 1 mL of 50% ACN:0.1% TFA aqueous solution under an ultrasound bath at 30 min. The sample was then centrifuged at 7000 rpm for 10 min, named H1, and the supernatant was diluted for 200 times and 0.5 mg/L $^{13}C_9{}^{15}N_1$-RCB-2 were added as IS for MALDI-MS to calculate the content of RCBs contained in 100 mg castor bean. The content of RCBs contained in 1 mg castor bean can be obtained after dividing by 100.

TEC factor estimation. After washing by Protocol E, tissue section from Ethiopia as an example was sprayed with 9 g/L CHCA, containing 0.5 mg/L $^{13}C_9{}^{15}N_1$-RCB-2. The region of interest (ROI) of the same pixels on the tissue section and ITO slide were selected for MSI acquisition. The TEC value was calculated by dividing the average intensity of $^{13}C_9{}^{15}N_1$-RCB-2 on the tissue area by the average intensity on the ITO slide area.

2 Supplementary Figures and Tables

2.1 Supplementary Figures

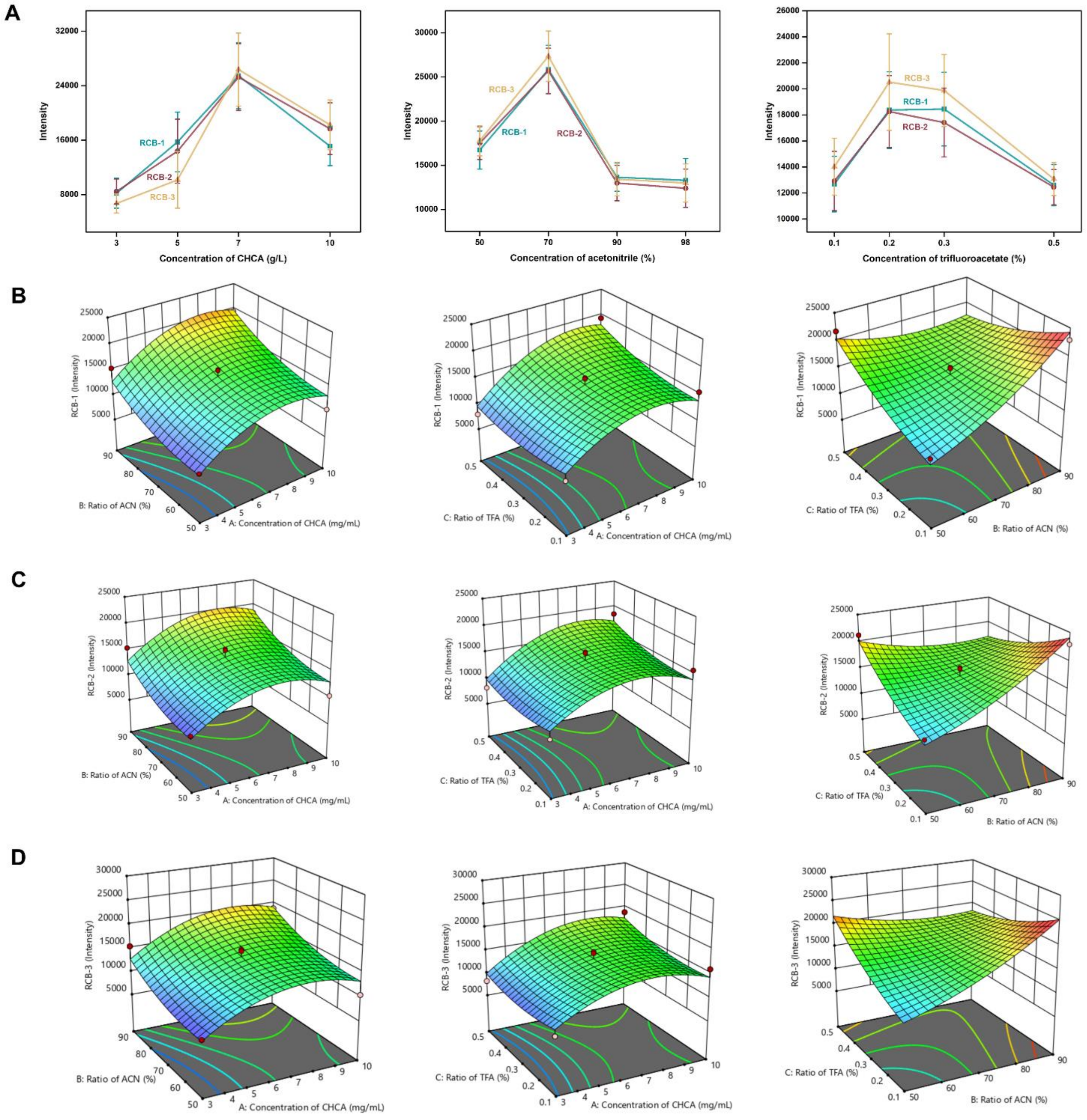

Figure S1. The appropriate range of CHCA concentration, ACN and TFA ratio by single factor experiment (A), the response surface plots showing the effects and mutual interactions of CHCA concentration, ACN ratio and TFA ratio on the intensity of RCB-1(B), RCB-2 (C), and RCB-3(D).

A

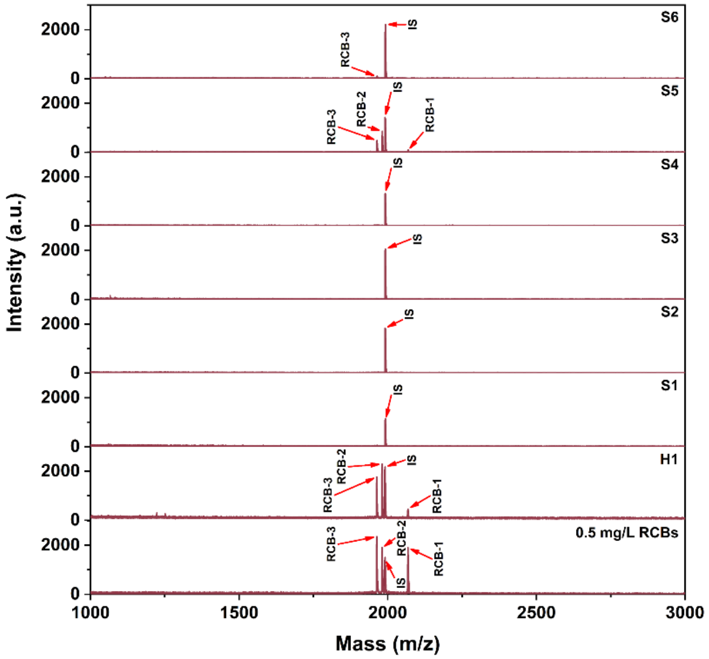

B

	Intensity ratio			Calculated concentration (mg/L)			Calculated content (ng)		
	RCB-1/IS	RCB-2/IS	RCB-3/IS	RCB-1	RCB-2	RCB-3	RCB-1	RCB-2	RCB-3
0.5 mg/L RCBs	1.2	1.1	1.4	/	/	/	/	/	/
100 mg castor bean homogenate solution diluted 200 times	0.2	1.0	0.8	0.1	0.4	0.3	/	/	/
S5 diluted 1.25 times	0.05	0.6	0.3	0.02	0.3	0.1	/	/	/
S6 diluted 1.25 times	/	/	0.04	/	/	0.01	/	/	/
100 mg castor bean homogenate solution	/	/	/	21.2	88.4	54.6	21.2E+3	88.4E+3	54.6E+3
S5 (Three consecutive tissue sections)	/	/	/	0.03	0.3	0.1	26.2	313.2	141.8
S6 (Three consecutive tissue sections)	/	/	/	/	/	0.02	/	/	16.9

C

	Content of RCBs (ng)								
	Per milligram Castor bean homogenate	S1	S2	S3	S4	S5	S6	Loss (%)	Recovery (%)
RCB-1	211.6	/	/	/	/	8.7	/	4.1%	95.9%
RCB-2	884.0	/	/	/	/	104.4	/	11.8%	88.2%
RCB-3	546.0	/	/	/	/	47.3	5.6	9.7%	90.3%

Figure S2. The recovery of RCBs after the washing procedure. (A) Mass Spectra determined by MALDI-MS of samples collected under each washing step of Protocol E. S1, S2, S3, S4, S5 and S6 represented the samples collected after washing tissue section with 70% isopropanol, 100% isopropanol, Carnoy's solution, 100% isopropanol, 0.1%TFA, 100% isopropanol for 30 s, respectively, H1 represented the 100 mg castor bean homogenate sample; (B) Concentration and content of RCBs in samples determined in MALDI-MS; (C) Loss content and recovery of RCBs after washing procedure by Protocol E.

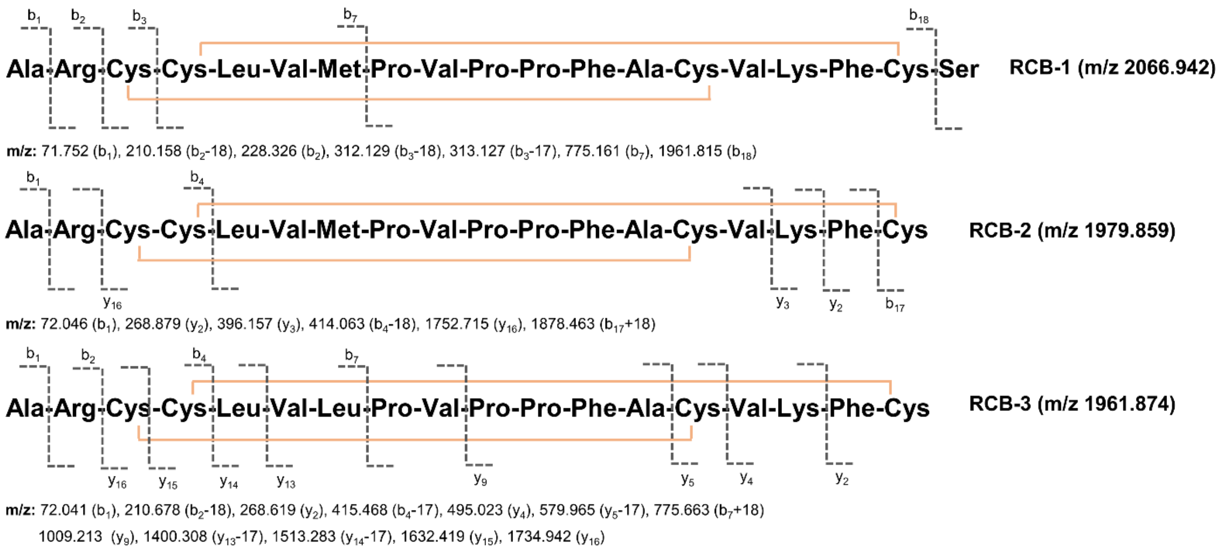

Figure S3. The *de novo* sequencing and disulfide bond connectivity of RCB-1 (Top), RCB-2 (Middle), and RCB-3 (Bottom).

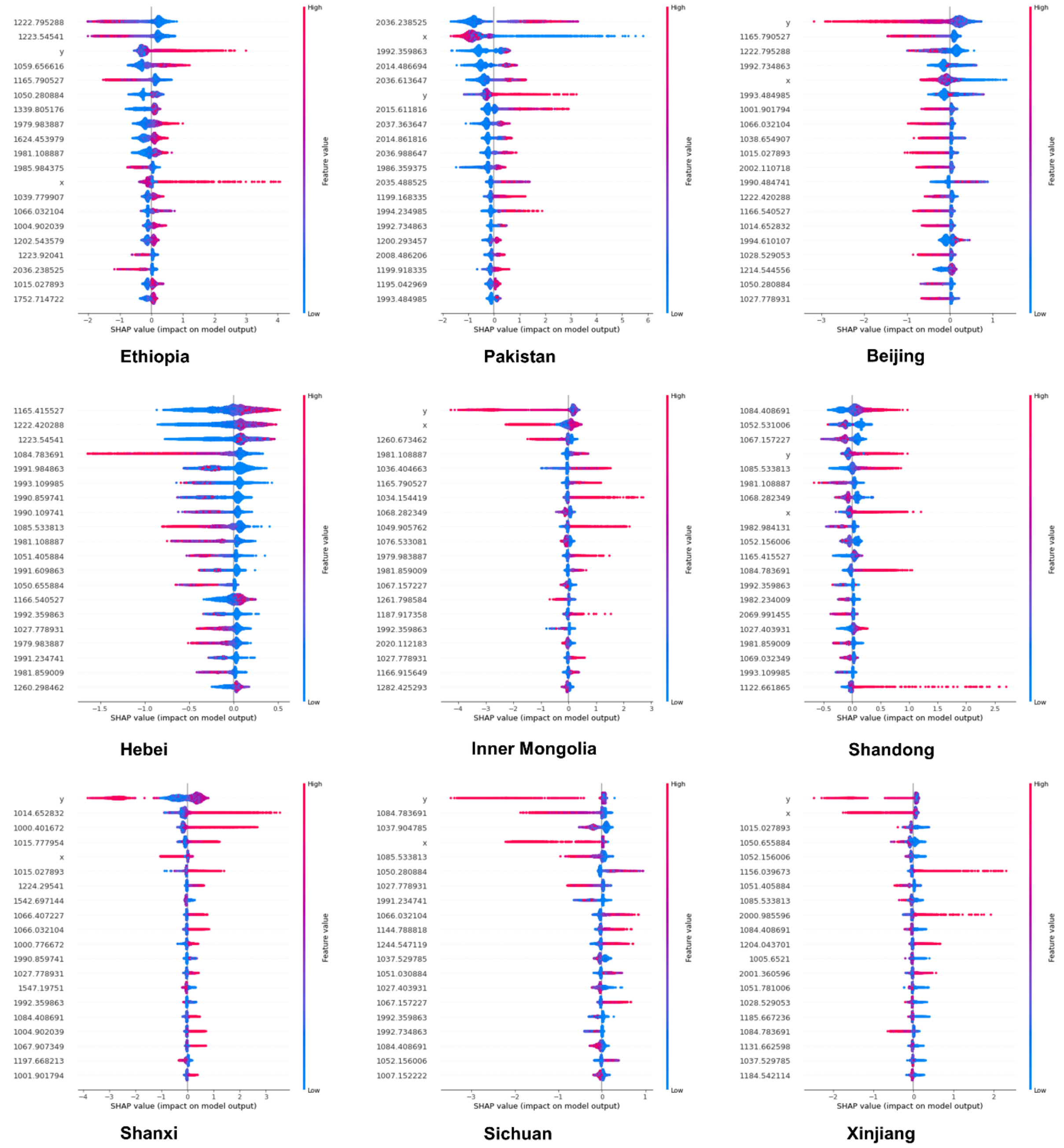

Figure S4. The feature density scatter plot of important features from nine different geographical sources. The top 20 features with SHAP values among the 2065-dimensional features was used for SHAP value visualization. Each row represented a feature, the abscissa was the SHAP value, a point represented a sample, and the wide segment represented a large number of samples clustered. The redder the dot, the higher the value of the feature itself, and the bluer the color, the lower the value of the feature itself.

2.2 Supplementary Tables

Table S1. Coded levels and actual values of the independent variables

Independent variables	Levels		
	-1	0	1
**Concentration of CHCA (mg/mL)**	3	6.5	10
**Ratio of ACN (%)**	50	70	90
**Ratio of TFA (%)**	0.1	0.3	0.5

Table S2. Experimental conditions for Box-Behnken experimental design and the corresponding results obtained for the intensity of RCBs

Run	Concentration of CHCA (mg/mL)	Ratio of ACN (%)	Ratio of TFA (%)	Intensity of RCB-1 (a.u.)		Intensity of RCB-2 (a.u.)		Intensity of RCB-3 (a.u.)	
				predicted	Actual	predicted	Actual	predicted	Actual
1	6.5	70	0.3	15302.0	14802.8	15464.1	15087.8	15614.2	14802.8
2	6.5	70	0.3	15302.0	14354.1	15464.1	14259.5	15614.2	14354.1
3	3	70	0.5	9561.3	7955.5	9820.7	8149.1	9882.7	7955.5
4	10	70	0.1	13865.4	15471.2	13966.1	15637.7	14207.1	15471.2
5	6.5	90	0.5	17748.8	16705.6	17207.4	16327.0	17328.1	16705.6
6	6.5	70	0.3	15302.0	14354.1	15464.1	14259.5	15614.2	14354.1
7	3	70	0.1	9943.0	8642.3	10221.2	8811.6	10075.7	8642.3
8	10	90	0.3	19684.5	19427.1	18900.6	18371.4	19111.5	19427.1
9	10	50	0.3	13089.9	10440.9	12887.7	10335.7	13103.9	10440.9
10	6.5	50	0.5	20355.1	21703.4	20058.8	21201.2	20976.2	21703.4
11	6.5	70	0.3	15302.0	16579.0	15464.1	16970.7	15614.2	16579.0
12	10	70	0.5	18043.5	19344.2	17084.1	18493.7	17416.9	19344.2
13	6.5	50	0.1	10028.9	11072.1	10474.1	11354.5	10429.6	11072.1
14	3	90	0.3	12709.4	15358.4	12758.0	15310.0	12661.2	15358.4
15	6.5	70	0.3	15302.0	16420.2	15464.1	16742.8	15614.2	16420.2
16	6.5	90	0.1	24278.7	22930.4	24074.5	22932.1	24857.9	22930.4
17	3	50	0.3	7660.5	7917.9	8021.9	8551.1	7888.6	7917.9

Table S3. Analysis of variance (ANOVA) for response surface quadratic model of RCB-2

Source	Sum of squares	Degree of freedom		Mean square	F-value	p-value
**Model**	2.63E+08	9	2.92E+07	5.96	0.014	significant
**A-Concentration of CHCA**	6.06E+07	1	6.06E+07	12.38	0.0097	
**B-Ratio of ACN**	5.78E+07	1	5.78E+07	11.8	0.0109	
**C-Ratio of TFA**	3.69E+06	1	3.69E+06	0.7545	0.4138	
**AB**	4.08E+05	1	4.08E+05	0.0833	0.7813	
**AC**	3.10E+06	1	3.10E+06	0.6324	0.4526	
**BC**	6.77E+07	1	6.77E+07	13.83	0.0075	
**A^2**	5.93E+07	1	5.93E+07	12.11	0.0103	
**B^2**	8.60E+06	1	8.60E+06	1.76	0.2265	
**C^2**	4.73E+06	1	4.73E+06	0.9673	0.3581	
**Residual**	3.43E+07	7	4.89E+06			
**Lack of Fit**	2.73E+07	3	9.10E+06	5.24	0.0717	not significant
**Pure Error**	6.95E+06	4	1.74E+06			
**Cor Total**	2.97E+08	16				

R^2=0.8846, $p<0.05$ is significant

Table S4. Experimental design of the orthogonal experiments with three factors and three levels $L_9(3^4)$ for the optimization of EFASS conditions

Test Number		Nitrogen pressure (P)	Spraying velocity（V）	Spraying time (T)
	1	0.1 MPa	10 µL/min	15 min
	2	0.1 MPa	20 µL/min	45 min
	3	0.1 MPa	30 µL/min	30 min
	4	0.2 MPa	10 µL/min	45 min
	5	0.2 MPa	20 µL/min	30 min
	6	0.2 MPa	30 µL/min	15 min
	7	0.3 MPa	10µL/min	30 min
	8	0.3 MPa	20 µL/min	15 min
	9	0.3 MPa	30 µL/min	45 min
RCB-1	k1	3.226	3.855	3.388
	k2	2.307	3.021	3.255
	k3	3.090	1.746	1.979
	Range	0.919	2.109	1.409
	The primary and secondary sequence	V>T>P		
	The optimal combination	Nitrogen pressure=0.1 MPa, spraying velocity= 10 µL/min, spraying time=15 min		
RCB-2	k1	10.332	13.880	11.746
	k2	5.653	8.102	10.152
	k3	10.741	4.743	4.827
	Range	5.088	9.137	6.919
	The primary and secondary sequence	V>T>P		
	The optimal combination	Nitrogen pressure=0.3 MPa, spraying velocity= 10 µL/min, spraying time=15 min		
RCB-3	k1	9.817	13.928	10.761
	k2	2.673	5.511	8.550
	k3	10.983	4.034	4.162
	Range	8.310	9.894	6.599
	The primary and secondary sequence	V>P>T		
	The optimal combination	Nitrogen pressure=0.3 MPa, spraying velocity= 10 µL/min, spraying time=15 min		

Table S5. LIPID MAPS database matching results of peaks in the range of m/z 1000~1200

Input Mass	Matched Mass	Delta	Name	Formula	Ion
1000.3513	1000.3028	0.0485	CoA 14:0	$C_{35}H_{62}N_7O_{17}P_3SNa$	$[M+Na]^+$
1000.7263	1000.7659	0.0396	Hex2Cer 44:2; O2	$C_{56}H_{105}NO_{13}$	$[M+H]^+$
1001.8514	1001.8532	0.0018	TG 63:8	$C_{66}H_{112}O_6$	$[M+H]^+$
1001.8514	1001.8508	0.0006	TG 61:5	$C_{64}H_{114}O_6Na$	$[M+Na]^+$
1002.2264	1002.2093	0.0171	CoA 11:2; O3	$C_{32}H_{52}N_7O_{20}P_3SNa$	$[M+Na]^+$
1002.2264	1002.2247	0.0017	CoA 12:1; O	$C_{33}H_{56}N_7O_{18}P_3SK$	$[M+K]^+$
1002.2264	1002.2611	0.0347	CoA 13:0	$C_{34}H_{60}N_7O_{17}P_3SK$	$[M+K]^+$
1003.7266	1003.7749	0.0483	TG 64:14	$C_{67}H_{102}O_6$	$[M+H]^+$
1003.7266	1003.7725	0.0459	TG 62:11	$C_{65}H_{104}O_6Na$	$[M+Na]^+$
1003.7266	1003.7151	0.0115	TG 61:12	$C_{64}H_{100}O_6K$	$[M+K]^+$
1005.6018	1005.5829	0.0189	PI 44:6	$C_{53}H_{91}O_{13}PK$	$[M+K]^+$
1006.3519	1006.3158	0.0361	CoA 15:1;O	$C_{36}H_{62}N_7O_{18}P_3S$	$[M+H]^+$
1006.3519	1006.3522	0.0003	CoA 16:0	$C_{37}H_{66}N_7O_{17}P_3S$	$[M+H]^+$
1007.8521	1007.9001	0.0480	TG 63:5	$C_{66}H_{118}O_6$	$[M+H]^+$
1007.8521	1007.8062	0.0459	TG 64:12	$C_{67}H_{106}O_6$	$[M+H]^+$
1007.8521	1007.8977	0.0456	TG 61:2	$C_{64}H_{120}O_6Na$	$[M+Na]^+$
1007.8521	1007.8038	0.0483	TG 62:9	$C_{65}H_{108}O_6Na$	$[M+Na]^+$
1007.8521	1007.8403	0.0118	TG 60:3	$C_{63}H_{116}O_6K$	$[M+K]^+$
1008.2271	1008.2351	0.0080	CoA 14:4;O	$C_{35}H_{54}N_7O_{18}P_3SNa$	$[M+Na]^+$
1009.7272	1009.7621	0.0349	TG 61:9	$C_{64}H_{106}O_6K$	$[M+K]^+$
1011.6024	1011.6298	0.0274	PI 44:3	$C_{53}H_{97}O_{13}PK$	$[M+K]^+$
1011.9775	1011.9314	0.0461	TG 63:3	$C_{66}H_{122}O_6$	$[M+H]^+$
1011.9775	1011.9290	0.0485	TG 61:0	$C_{64}H_{124}O_6Na$	$[M+Na]^+$
1012.3525	1012.3028	0.0497	CoA 15:1	$C_{36}H_{62}N_7O_{17}P_3SNa$	$[M+Na]^+$
1012.7275	1012.7061	0.0214	Hex2Cer 42:1;O2	$C_{54}H_{103}NO_{13}K$	$[M+K]^+$
1013.8527	1013.8532	0.0005	TG 64:9	$C_{67}H_{112}O_6$	$[M+H]^+$
1013.8527	1013.8508	0.0019	TG 62:6	$C_{65}H_{114}O6Na$	$[M+Na]^+$
1013.8527	1013.8873	0.0346	TG 60:0	$C_{63}H_{122}O_6K$	$[M+K]^+$
1014.2277	1014.2093	0.0184	CoA 12:3;O3	$C_{33}H_{52}N_7O_{20}P_3SNa$	$[M+Na]^+$
1014.2277	1014.2611	0.0334	CoA 14:1	$C_{35}H_{60}N_7O_{17}P_3SK$	$[M+K]^+$
1015.7279	1015.7725	0.0446	TG 63:12	$C_{66}H_{104}O_6Na$	$[M+Na]^+$
1015.7279	1015.7151	0.0128	TG 62:13	$C_{65}H_{100}O_6K$	$[M+K]^+$
1017.9781	1017.9784	0.0003	TG 63:0	$C_{66}H_{128}O_6$	$[M+H]^+$
1018.3531	1018.3522	0.0009	CoA 17:1	$C_{38}H_{66}N_7O_{17}P_3S$	$[M+H]^+$
1018.7282	1018.7259	0.0023	PC 54:12	$C_{62}H_{100}NO_8P$	$[M+H]^+$
1019.8533	1019.9001	0.0468	TG 64:6	$C_{67}H_{118}O6$	$[M+H]^+$
1019.8533	1019.8977	0.0444	TG 62:3	$C_{65}H_{120}O_6Na$	$[M+Na]^+$
1019.8533	1019.8038	0.0495	TG 63:10	$C_{66}H_{108}O_6Na$	$[M+Na]^+$
1019.8533	1019.8403	0.0130	TG 61:4	$C_{64}H_{116}O_6K$	$[M+K]^+$
1021.7285	1021.7256	0.0029	TG 64:16	$C_{67}H_{98}O_6Na$	$[M+Na]^+$
1021.7285	1021.7621	0.0336	TG 62:10	$C_{65}H_{106}O_6K$	$[M+K]^+$
1023.9788	1023.9314	0.0474	TG 64:4	$C_{67}H_{122}O_6$	$[M+H]^+$
1023.9788	1023.9290	0.0498	TG 62:1	$C_{65}H_{124}O_6Na$	$[M+Na]^+$
1023.9788	1024.0018	0.0230	FA 67:1;O2	$C_{67}H_{132}O_4Na$	$[M+Na]^+$
1024.7289	1024.7635	0.0346	Hex2Cer 44:1;O2	$C_{56}H_{107}NO_{13}Na$	$[M+Na]^+$
1025.8540	1025.8532	0.0008	TG 65:10	$C_{68}H_{112}O_6$	$[M+H]^+$
1025.8540	1025.8508	0.0032	TG 63:7	$C_{66}H_{114}O_6Na$	$[M+Na]^+$
1025.8540	1025.8873	0.0333	TG 61:1	$C_{64}H_{122}O_6K$	$[M+K]^+$
1026.6040	1026.6101	0.0061	MIPC 36:0;O4	$C_{48}H_{94}NO_{18}PNa$	$[M+Na]^+$
1026.6040	1026.5891	0.0149	MIPC 36:0;O3	$C_{48}H_{94}NO_{17}PK$	$[M+K]^+$
1027.7291	1027.7749	0.0458	TG 66:16	$C_{69}H_{102}O_6$	$[M+H]^+$
1027.7291	1027.7725	0.0434	TG 64:13	$C_{67}H_{104}O_6Na$	$[M+Na]^+$
1029.9794	1029.9784	0.0010	TG 64:1	$C_{67}H_{128}O_6$	$[M+H]^+$
1030.3545	1030.3522	0.0023	CoA 18:2	$C_{39}H_{66}N_7O_{17}P_3S$	$[M+H]^+$
1030.3545	1030.3134	0.0411	CoA 15:0;O	$C_{36}H_{64}N_7O_{18}P_3SNa$	$[M+Na]^+$
1031.8546	1031.9001	0.0455	TG 65:7	$C_{68}H_{118}O_6$	$[M+H]^+$
1031.8546	1031.8062	0.0484	TG 66:14	$C_{69}H_{106}O_6$	$[M+H]^+$
1031.8546	1031.8977	0.0431	TG 63:4	$C_{66}H_{120}O_6Na$	$[M+Na]^+$
1031.8546	1031.8403	0.0143	TG 62:5	$C_{65}H_{116}O_6K$	$[M+K]^+$
1032.2296	1032.2717	0.0421	CoA 14:0;O	$C_{35}H_{62}N_7O_{18}P_3SK$	$[M+K]^+$
1033.7299	1033.7621	0.0322	TG 63:11	$C_{66}H_{106}O_6K$	$[M+K]^+$

1035.9801	1035.9314	0.0487	TG 65:5	$C_{68}H_{122}O_6$	$[M+H]^+$
1036.3551	1036.3627	0.0076	CoA 17:0;O	$C_{38}H_6 8N_7O_{18}P_3S$	$[M+H]^+$
1037.8552	1037.8532	0.0020	TG 66:11	$C_{69}H_{112}O_6$	$[M+H]^+$
1037.8552	1037.8508	0.0044	TG 64:8	$C_{67}H_{114}O_6Na$	$[M+Na]^+$
1037.8552	1037.8873	0.0321	TG 62:2	$C_{65}H_{122}O_6K$	$[M+K]^+$
1038.6053	1038.6465	0.0412	MIPC 38:0;O3	$C_{50}H_{98}NO_{17}PNa$	$[M+Na]^+$
1038.6053	1038.6255	0.0202	MIPC 38:0;O2	$C_{50}H_{98}NO_{16}PK$	$[M+K]^+$
1038.9803	1038.9553	0.0250	ACer 66:1;O2	$C_{66}H_{129}NO_4K$	$[M+K]^+$
1039.7305	1039.7151	0.0154	TG 64:15	$C_{67}H_{100}O_6K$	$[M+K]^+$
1041.9807	1041.9784	0.0023	TG 65:2	$C_{68}H_{128}O_6$	$[M+H]^+$
1042.3557	1042.3158	0.0399	CoA 18:4;O	$C_{39}H_{62}N_7O_{18}P_3S$	$[M+H]^+$
1042.3557	1042.3134	0.0423	CoA 16:1;O	$C_{37}H_{64}N_7O_{18}P_3SNa$	$[M+Na]^+$
1042.3557	1042.3497	0.0060	CoA 17:0	$C_{38}H_{68}N_7O_{17}P_3SNa$	$[M+Na]^+$
1042.7308	1042.7530	0.0222	Hex2Cer 44:0;O2	$C_{56}H_{109}NO_{13}K$	$[M+K]^+$
1043.8560	1043.9001	0.0441	TG 66:8	$C_{69}H_{118}O_6$	$[M+H]^+$
1043.8560	1043.8977	0.0417	TG 64:5	$C_{67}H_{120}O_6Na$	$[M+Na]^+$
1043.8560	1043.8403	0.0157	TG 63:6	$C_{66}H_{116}O_6K$	$[M+K]^+$
1044.2310	1044.2717	0.0407	CoA 15:1;O	$C_{36}H_{62}N_7O_{18}P_3SK$	$[M+K]^+$
1045.7311	1045.7256	0.0055	TG 66:18	$C_{69}H_{98}O_6Na$	$[M+Na]^+$
1045.7311	1045.7621	0.0310	TG 64:12	$C_{67}H_{106}O_6K$	$[M+K]^+$
1046.1062	1046.0824	0.0238	FA 70:0;O2	$C_{70}H_{140}O_4$	$[M+H]^+$
1047.9813	1047.9314	0.0499	TG 66:6	$C_{69}H_{122}O_6$	$[M+H]^+$
1048.3564	1048.3627	0.0063	CoA 18:1;O	$C_{39}H_{68}N_7O_{18}P_3S$	$[M+H]^+$
1048.3564	1048.3991	0.0427	CoA 19:0	$C_{40}H_{72}N_7O_{17}P_3S$	$[M+H]^+$
1049.8566	1049.8508	0.0058	TG 65:9	$C_{68}H_{114}O_6Na$	$[M+Na]^+$
1049.8566	1049.8873	0.0307	TG 63:3	$C_{66}H_{122}O_6K$	$[M+K]^+$
1051.7317	1051.7725	0.0408	TG 66:15	$C_{69}H_{104}O_6Na$	$[M+Na]^+$
1052.8568	1052.8383	0.0185	PC 52:0	$C_{60}H_{120}NO_8PK$	$[M+K]^+$
1053.9819	1053.9784	0.0035	TG 66:3	$C_{69}H_{128}O_6$	$[M+H]^+$
1053.9819	1053.9760	0.0059	TG 64:0	$C_{67}H_{130}O_6Na$	$[M+Na]^+$
1054.3571	1054.3522	0.0049	CoA 20:4	$C_{41}H_{66}N_7O_{17}P_3S$	$[M+H]^+$
1054.3571	1054.3497	0.0074	CoA 18:1	$C_{39}H_{68}N_7O_{17}P_3SNa$	$[M+Na]^+$
1055.4822	1055.4634	0.0188	PIP2 37:4	$C_{46}H_{83}O_{19}P_3Na$	$[M+Na]^+$
1055.8572	1055.8977	0.0405	TG 65:6	$C_{68}H_{120}O_6Na$	$[M+Na]^+$
1055.8572	1055.8403	0.0169	TG 64:7	$C_{67}H_{116}O_6K$	$[M+K]^+$
1058.1074	1058.0824	0.0250	FA 71:1;O2	$C_{71}H_{140}O_4$	$[M+H]^+$
1059.9827	1060.0253	0.0426	TG 66:0	$C_{69}H_{134}O_6$	$[M+H]^+$
1060.3577	1060.3991	0.0414	CoA 20:1	$C_{41}H_{72}N_7O_{17}P_3S$	$[M+H]^+$
1060.7327	1060.6907	0.0420	MIPC 40:0;O4	$C_{52}H_{102}NO_{18}P$	$[M+H]^+$
1061.8578	1061.8508	0.0070	TG 66:10	$C_{69}H_{114}O_6Na$	$[M+Na]^+$
1061.8578	1061.8873	0.0295	TG 64:4	$C_{67}H_{122}O_6K$	$[M+K]^+$
1063.7330	1063.7151	0.0179	TG 66:17	$C_{69}H_{100}O_6K$	$[M+K]^+$
1065.9833	1065.9760	0.0073	TG 65:1	$C_{68}H_{130}O_6Na$	$[M+Na]^+$
1066.3583	1066.3134	0.0449	CoA 18:3;O	$C_{39}H_{64}N_7O_{18}P_3SNa$	$[M+Na]^+$
1067.8585	1067.8977	0.0392	TG 66:7	$C_{69}H_{120}O_6Na$	$[M+Na]^+$
1067.8585	1067.8403	0.0182	TG 65:8	$C_{68}H_{116}O_6K$	$[M+K]^+$
1069.7336	1069.7621	0.0285	TG 66:14	$C_{69}H_{106}O_6K$	$[M+K]^+$
1071.9839	1072.0042	0.0203	CE 46:3;O2	$C_{73}H_{130}O_4$	$[M+H]^+$
1072.3590	1072.3627	0.0037	CoA 20:3;O	$C_{41}H_{68}N_7O_{18}P_3S$	$[M+H]^+$
1072.3590	1072.3603	0.0013	CoA 18:0;O	$C_{39}H_{70}N_7O_{18}P_3SNa$	$[M+Na]^+$
1072.3590	1072.3393	0.0197	CoA 18:0	$C_{39}H_{70}N_7O_{17}P_3SK$	$[M+K]^+$
1072.7340	1072.7271	0.0069	MIPC 42:0;O3	$C_{54}H_{106}NO_{17}P$	$[M+H]^+$
1073.8591	1073.8873	0.0282	TG 65:5	$C_{68}H_{122}O_6K$	$[M+K]^+$
1077.9846	1077.9760	0.0086	TG 66:2	$C_{69}H_{130}O_6Na$	$[M+Na]^+$
1078.3596	1078.3522	0.0074	CoA 22:6	$C_{43}H_{66}N_7O_{17}P_3S$	$[M+H]^+$
1078.3596	1078.3497	0.0099	CoA 20:3	$C_{41}H_{68}N_7O_{17}P_3SNa$	$[M+Na]^+$
1078.7346	1078.7141	0.0205	MIPC 42:0;O2	$C_{54}H_{106}NO_{16}PNa$	$[M+Na]^+$
1079.8597	1079.8403	0.0194	TG 66:9	$C_{69}H_{116}O_6K$	$[M+K]^+$
1080.2349	1080.2717	0.0368	CoA 18:4;O	$C_{39}H_{62}N_7O_{18}P_3SK$	$[M+K]^+$
1082.1100	1082.0800	0.0300	FA 71:0;O2	$C_{71}H_{142}O_4Na$	$[M+Na]^+$
1083.9852	1083.9655	0.0197	TG 65:0	$C_{68}H_{132}O_6K$	$[M+K]^+$
1084.3602	1084.3603	0.0001	CoA 19:1;O	$C_{40}H_{70}N_7O_{18}P_3SNa$	$[M+Na]^+$
1084.3602	1084.3967	0.0365	CoA 20:0	$C_{41}H_{74}N_7O_{17}P_3SNa$	$[M+Na]^+$
1084.3602	1084.3393	0.0209	CoA 19:1	$C_{40}H_{70}N_7O_{17}P_3SK$	$[M+K]^+$
1084.7352	1084.7635	0.0283	MIPC 44:0;O2	$C_{56}H_{110}NO_{16}P$	$[M+H]^+$
1084.7352	1084.7131	0.0221	PC 56:12	$C_{64}H_{104}NO_8PK$	$[M+K]^+$

1085.8604	1085.8873	0.0269	TG 66:6	$C_{69}H_{122}O_6K$	$[M+K]^+$
1088.1106	1088.1294	0.0188	FA 73:0;O2	$C_{73}H_{146}O_4$	$[M+H]^+$
1090.3608	1090.3134	0.0474	CoA 20:5;O	$C_{41}H_{64}N_7O_{18}P_3SNa$	$[M+Na]^+$
1090.7360	1090.7601	0.0241	PC 56:9	$C_{64}H_{110}NO_8PK$	$[M+K]^+$
1094.1113	1094.0800	0.0313	FA 72:1;O2	$C_{72}H_{142}O_4Na$	$[M+Na]^+$
1095.9865	1095.9655	0.0210	TG 66:1	$C_{69}H_{132}O_6K$	$[M+K]^+$
1096.3616	1096.3627	0.0011	CoA 22:5;O	$C_{43}H_{68}N_7O_{18}P_3S$	$[M+H]^+$
1096.3616	1096.3991	0.0375	CoA 23:4	$C_{44}H_{72}N_7O_{17}P_3S$	$[M+H]^+$
1096.3616	1096.3603	0.0013	CoA 20:2;O	$C_{41}H_{70}N_7O_{18}P_3SNa$	$[M+Na]^+$
1096.3616	1096.3393	0.0223	CoA 20:2	$C_{41}H_{70}N_7O_{17}P_3SK$	$[M+K]^+$
1098.6118	1098.6466	0.0348	MIPC 40:0;O4	$C_{52}H_{102}NO_{18}PK$	$[M+K]^+$
1100.1119	1100.1294	0.0175	FA 74:1;O2	$C_{74}H_{146}O_4$	$[M+H]^+$
1102.3622	1102.4097	0.0475	CoA 22:2;O	$C_{43}H_{74}N_7O_{18}P_3S$	$[M+H]^+$
1102.3622	1102.3497	0.0125	CoA 22:5	$C_{43}H_{68}N_7O_{17}P_{3S}Na$	$[M+Na]^+$
1102.3622	1102.3499	0.0123	CoA 19:0;O	$C_{40}H_{72}N_7O_{18}P_3SK$	$[M+K]^+$
1106.4877	1106.4410	0.0467	CoA 22:0;O	$C_{43}H_{78}N_7O_{18}P_3S$	$[M+H]^+$
1108.3628	1108.3991	0.0363	CoA 24:5	$C_{45}H_{72}N_7O_{17}P_3S$	$[M+H]^+$
1108.3628	1108.3967	0.0339	CoA 22:2	$C_{43}H_{74}N_7O_{17}P_3SNa$	$[M+Na]^+$
1112.1133	1112.0696	0.0437	FA 72:0;O2	$C_{72}H_{144}O_4K$	$[M+K]^+$
1114.3635	1114.3499	0.0136	CoA 20:1;O	$C_{41}H_{72}N_7O_{18}P_3SK$	$[M+K]^+$
1118.4889	1118.4774	0.0115	CoA 24:0	$C_{45}H_{82}N_7O_{17}P_3S$	$[M+H]^+$
1120.3641	1120.3627	0.0014	CoA 24:7;O	$C_{45}H_{68}N_7O_{18}P_3S$	$[M+H]^+$
1120.3641	1120.3393	0.0248	CoA 22:4	$C_{43}H_{70}N_7O_{17}P_3SK$	$[M+K]^+$
1124.1145	1124.1270	0.0125	FA 74:0;O2	$C_{74}H_{148}O_4Na$	$[M+Na]^+$
1124.1145	1124.0696	0.0449	FA 73:1;O2	$C_{73}H_{144}O_4K$	$[M+K]^+$
1126.3647	1126.4073	0.0426	CoA 22:1;O	$C_{43}H_{76}N_7O_{18}P_3SNa$	$[M+Na]^+$
1126.3647	1126.3497	0.0150	CoA 24:7	$C_{45}H_{68}N_7O_{17}P_3SNa$	$[M+Na]^+$
1126.3647	1126.3863	0.0216	CoA 22:1	$C_{43}H_{76}N_7O_{17}P_3SK$	$[M+K]^+$
1130.4902	1130.4410	0.0492	CoA 24:2;O	$C_{45}H_{78}N_7O_{18}P_3S$	$[M+H]^+$
1132.3654	1132.3967	0.0313	CoA 24:4	$C_{45}H_{74}N_7O_{17}P_3SNa$	$[M+Na]^+$
1136.1158	1136.1270	0.0112	FA 75:1;O2	$C_{75}H_{148}O_4Na$	$[M+Na]^+$
1137.9910	1137.9914	0.0004	CE 48:3;O2	$C_{75}H_{134}O_4K$	$[M+K]^+$
1138.7411	1138.7353	0.0058	MIPC 44:0;O4	$C_{56}H_{110}NO_{18}PNa$	$[M+Na]^+$
1138.7411	1138.7143	0.0268	MIPC 44:0;O3	$C_{56}H_{110}NO_{17}PK$	$[M+K]^+$
1144.3667	1144.3603	0.0064	CoA 24:6;O	$C_{45}H_{70}N_7O_{18}P_3SNa$	$[M+Na]^+$
1144.3667	1144.3969	0.0302	CoA 22:0;O	$C_{43}H_{78}N_7O_{18}P_3SK$	$[M+K]^+$
1144.3667	1144.3393	0.0274	CoA 24:6	$C_{45}H_{70}N_7O_{17}P_3SK$	$[M+K]^+$
1144.7417	1144.7846	0.0429	MIPC 46:0;O4	$C_{58}H_{114}NO_{18}P$	$[M+H]^+$
1150.7423	1150.7717	0.0294	MIPC 46:0;O3	$C_{58}H_{114}NO_{17}PNa$	$[M+Na]^+$
1150.7423	1150.7507	0.0084	MIPC 46:0;O2	$C_{58}H_{114}NO_{16}PK$	$[M+K]^+$
1154.1177	1154.1166	0.0011	FA 75:0;O2	$C_{75}H_{150}O_4K$	$[M+K]^+$
1158.2432	1158.2076	0.0356	FA 78:0;O2	$C_{78}H_{156}O_4$	$[M+H]^+$
1160.1184	1160.1634	0.0450	FA 78:2;O	$C_{78}H_{152}O_3Na$	$[M+Na]^+$
1160.4934	1160.4879	0.0055	CoA 26:1;O	$C_{47}H_{84}N_7O_{18}P_3S$	$[M+H]^+$
1162.3687	1162.3499	0.0188	CoA 24:5;O	$C_{45}H_{72}N_7O_{18}P_3SK$	$[M+K]^+$
1166.1190	1166.1166	0.0024	FA 76:1;O2	$C_{76}H_{150}O_4K$	$[M+K]^+$
1166.4940	1166.4749	0.0191	CoA 26:1	$C_{47}H_{84}N_7O_{17}P_3SNa$	$[M+Na]^+$
1168.3693	1168.3969	0.0276	CoA 24:2;O	$C_{45}H_{78}N_7O_{18}P_3SK$	$[M+K]^+$
1170.2445	1170.2076	0.0369	FA 79:1;O2	$C_{79}H_{156}O_4$	$[M+H]^+$
1178.1204	1178.0800	0.0404	CE 52:3;O2	$C_{79}H_{142}O_4Na$	$[M+Na]^+$
1184.4960	1184.4855	0.0105	CoA 26:0;O	$C_{47}H_{86}N_7O_{18}P_3SNa$	$[M+Na]^+$
1184.4960	1184.4645	0.0315	CoA 26:0	$C_{47}H_{86}N_7O_{17}P_3SK$	$[M+K]^+$
1194.2471	1194.2052	0.0419	FA 79:0;O2	$C_{79}H_{158}O_4Na$	$[M+Na]^+$
1196.1222	1196.1635	0.0413	FA 78:0;O2	$C_{78}H_{156}O_4K$	$[M+K]^+$
1196.4973	1196.5219	0.0246	CoA 28:0	$C_{49}H_{90}N_7O_{17}P_3SNa$	$[M+Na]^+$
1200.2477	1200.2546	0.0069	FA 81:0;O2	$C_{81}H_{162}O_4$	$[M+H]^+$

Table S6. TEC value on tissue section of castor bean

	Intensity of $^{13}C_9{}^{15}N_1$-RCB-2 (a.u.)		TEC Value
	**Tissue section**	**ITO slide**	
**ROI_1**	2.9	305.5	0.009
**ROI_2**	4.7	371.0	0.013
**ROI_3**	6.0	516.3	0.012
**Mean TEC Value**			0.011
**RSD (%)**			14.4%
